# Inhibition of Carbohydrate Metabolism Potentiated by the Therapeutic Effects of Oxidative Phosphorylation Inhibitors in Colon Cancer Cells

**DOI:** 10.3390/cancers16071399

**Published:** 2024-04-02

**Authors:** Lichao Guo, Baochen Zhang, Wen Zhang, Yanqi Xie, Xi Chen, Xueke Sun, David S. Watt, Chunming Liu, H. Peter Spielmann, Xifu Liu

**Affiliations:** 1 Ministry of Education Key Laboratory of Molecular and Cellular Biology, Hebei Key Laboratory and Center for Drug Innovation and Discovery, College of Life Sciences, Hebei Normal University, Shijiazhuang 050024, China; 2 Department of Molecular and Cellular Biochemistry, College of Medicine, University of Kentucky, Lexington, KY 40536, USA; 3 Lucille Parker Markey Cancer Center, University of Kentucky, Lexington, KY 40536, USA

**Keywords:** isoflavonoid, colorectal cancer, OXPHOS, glycolysis, mitochondria complex I, ketogenic diet

## Abstract

**Simple Summary:**

Glycolysis and oxidative phosphorylation play important roles in the progression and growth of cancers. The development of natural products and their semisynthetic derivatives for cancer treatment is a longstanding focus of our research interests. We developed compounds known as diaminobutoxy-substituted isoflavonoids (DBIs) that effectively stimulated Adenosine 5′ Monophosphate-activated Protein Kinase (AMPK) and suppressed the growth of colorectal cancer cells by specifically targeting mitochondrial complex I. We now report a new DBI analog, namely, DBI-2, with promising properties for cancer treatment. The combination of DBI-2 and BAY-876, a glucose transporter 1 inhibitor, exhibited synergistic effects on colorectal cancer cells. Furthermore, the therapeutic effectiveness of DBI-2 in colorectal cancer cell xenograft mouse models was enhanced by implementing a ketogenic diet, an outcome that indicated this drug/diet combination is a potentially promising combination strategy for cancer therapy.

**Abstract:**

Cancer cells undergo a significant level of “metabolic reprogramming” or “remodeling” to ensure an adequate supply of ATP and “building blocks” for cell survival and to facilitate accelerated proliferation. Cancer cells preferentially use glycolysis for ATP production (the Warburg effect); however, cancer cells, including colorectal cancer (CRC) cells, also depend on oxidative phosphorylation (OXPHOS) for ATP production, a finding that suggests that both glycolysis and OXPHOS play significant roles in facilitating cancer progression and proliferation. Our prior studies identified a semisynthetic isoflavonoid, DBI-1, that served as an AMPK activator targeting mitochondrial complex I. Furthermore, DBI-1 and a glucose transporter 1 (GLUT1) inhibitor, BAY-876, synergistically inhibited CRC cell growth in vitro and in vivo. We now report a study of the structure–activity relationships (SARs) in the isoflavonoid family in which we identified a new DBI-1 analog, namely, DBI-2, with promising properties. Here, we aimed to explore the antitumor mechanisms of DBIs and to develop new combination strategies by targeting both glycolysis and OXPHOS. We identified DBI-2 as a novel AMPK activator using an AMPK phosphorylation assay as a readout. DBI-2 inhibited mitochondrial complex I in the Seahorse assays. We performed proliferation and Western blotting assays and conducted studies of apoptosis, necrosis, and autophagy to corroborate the synergistic effects of DBI-2 and BAY-876 on CRC cells in vitro. We hypothesized that restricting the carbohydrate uptake with a KD would mimic the effects of GLUT1 inhibitors, and we found that a ketogenic diet significantly enhanced the therapeutic efficacy of DBI-2 in CRC xenograft mouse models, an outcome that suggested a potentially new approach for combination cancer therapy.

## 1. Introduction

Colorectal cancer (CRC) is responsible for a significant number of global cancer-related fatalities as evidenced by 1.85 million reported cases and 850,000 annual deaths. At the present time, CRC accounts for 6.1% of global cancer incidence and 9.2% of cancer-related mortality [1,2]. Despite advances in treatments for metastatic disease, the five-year survival rate for patients with late-stage CRC (i.e., stage IV or metastatic CRC) is only 14.2% [3,4], and current projections suggest that the societal burden of CRC will increase by 60% by 2030 [5]. Efforts to mitigate this disease will require new strategies, including those that involve a combination of two or more drugs or a combination of different types of therapies.

The reprogramming of energy metabolism is an important characteristic of cancer cells [6]. The metabolic reprogramming observed in cancer cells differs from that of normal cells by encompassing an enhanced glycolytic pathway, augmented glutaminolysis flux, upregulated amino acid and lipid metabolism, facilitated mitochondrial biogenesis, initiation of the pentose phosphate pathway, and synthesis of macromolecules [7]. Cancer cells utilize carbohydrates, fats, and proteins to generate intermediates that enter glycolysis and the TCA cycle and that drive the electron transport chain (ETC) [8]. Collectively, these processes generate energy-rich ATP and/or GTP either directly, as seen in glycolysis and the TCA cycle, or indirectly, as in the conversion of NADH in the ETC in the inner mitochondrial membrane [9] to ATP [8]. In normal cells in the presence of oxygen, glucose is metabolized to pyruvate in the cytosol and pyruvate is translocated to the mitochondrial matrix and further metabolized through the tricarboxylic acid (TCA) cycle to generate ATP/GTP and NADH. The subsequent oxidative phosphorylation (OXPHOS) of NADH in the inner mitochondrial membrane provides a rich source of ATP. In the absence of oxygen, glycolysis is favored and relatively less pyruvate is allocated to the oxygen-consuming mitochondria. In cancer cells, glycolysis activity undergoes a marked increase even in the presence of oxygen, a phenomenon known as the Warburg effect [10,11,12]. However, ATP production from glycolysis is less efficient, and cancer cells, particularly cancer stem cells, must rely on OXPHOS to provide ATP for survival and growth [8,13,14]. 

The AMPK is a serine/threonine kinase that is highly conserved throughout evolution and that functions as a pivotal metabolic sensor and regulator of cellular energy homeostasis [15]. AMPK is phosphorylated and activated when cellular energy levels are reduced due to circumstances, such as hypoxia and a lack of nutrients [16]. AMPK thus plays a crucial role in the regulation of various metabolic pathways, including those involved in carbohydrate, lipid, and protein metabolism [17,18]. As a stress-response molecule, AMPK is intricately linked to tumor-suppressive functions through the inhibition of Akt and the downstream mammalian target of rapamycin (mTOR) activity that thereby induces growth suppression and cell cycle arrest [19]. 

BAY-876, a highly selective inhibitor of GLUT1, decreases the uptake of glucose by cancer cells and consequently leads to decreased ATP production [20]. However, in a mouse model of an ovarian cancer xenograft, the effective dose of BAY-876 (4.5 mg/kg/day) induced significant weight loss, a finding that indicated that BAY-876 had a narrow therapeutic window [21]. We hypothesized that the co-administration of other inhibitors targeting energy metabolism would enhance the cancer therapeutic efficacy of BAY-876. AMPK activation that is triggered when cellular energy levels are reduced allowed us to use an AMPK phosphorylation assay as a readout to screen the potential energy metabolism inhibitors.

As part of our continued interest in exploring semisynthetic derivatives of natural products as potentially useful antineoplastic agents, we identified a family of isoflavonoids, including the diaminobutoxy-substituted isoflavonoid (DBI-1), as AMPK activators. We explored combinatorial approaches for cancer treatment and found that DBI-1 had a synergistic effect with BAY-876 in inhibiting CRC cell growth in vitro and in vivo [22]. Based on this finding, we also hypothesized that the calorie-restricted ketogenic diet (KD) would mimic the effects of GLUT1 inhibitors and reduce glucose uptake by cancer cells and would further enhance the anticancer effect of OXPHOS inhibitors. The KD dietary regimen utilizes high fat content, low carbohydrate intake, and limited protein consumption. By significantly restricting carbohydrates, it simulates a fasting state, leading to reduced blood glucose levels and the production of ketone bodies [23,24]. The ketone bodies in turn serve as viable and alternative sources of energy for normal cells that possess functional mitochondria, but these ketone bodies serve no purpose in tumor cells that have dysfunctional mitochondria and that cannot compensate for glucose limitations by utilizing ketone bodies as an alternative fuel source [25,26,27,28]. Decreased ketogenesis is a signature of CRC, and consequently, a KD represses CRC growth [29]. A KD has previously served as a safe and achievable component in a treatment strategy for certain types of cancers [30,31]. Recent research demonstrated that a ketogenic diet had anticancer effects in DMH-induced CRC in rats [32]. These findings prompted our exploration of the synergistic effects of targeting metabolic reprogramming in colorectal cancer through a combination of drug and diet, which would increase the therapeutic efficacy and overcome drug resistance.

## 2. Materials and Methods

### 2.1. Chemistry

The chemicals and solvents were purchased from Sigma Aldrich (St. Louis, MO, USA), except for the following: resorcinol and 4-bromophenylacetic acid obtained from Acros Organic (Carlsbad, CA, USA), 1,4-dibromobutane obtained from Oakwood Chemical (Estill, SC, USA), 1,8-dibromooctane obtained from Rhawn (Shanghai, China), sodium iodide obtained from Fisher Scientific (Waltham, MA, USA), and piperazine and 1-(2-hydroxyethyl)piperazine obtained from (J&K Scientific (Shijiazhuang, Hebei, China).

The nuclear magnetic resonance spectra were measured in CDCl_3_ or DMSO-d_6_ using Bruker instruments (^1^H, 600; ^13^C, 151Mz; Bruker, Inc., Billerica, MA, USA). The LTQ-Orbitrap Velos mass spectrometer was utilized to acquire the high-resolution electrospray ionization (ESI) mass spectra (Thermo Fisher Scientific, Waltham, MA, USA). The FT resolution was set at 100,000 (at 400 *m/z*). The samples were introduced by means of direct infusion utilizing a syringe pump operating at a flow rate of 5 µL/min.

DBI-1 and DBI-2 were synthesized using the methods described previously [22] and fully characterized according to standard conventions (Supplementary Materials File S1).

### 2.2. Biology Materials

Antibodies used in Western blotting: p-AMPK (#2535, CST), AMPK (#2532, CST), p-ACC (#11818, CST), ACC (#3676, CST), p-P70S6K (#9234, CST), P70S6K (#2708, CST), p-S6 (#4858, CST), S6 (#2317, CST), Axin2 (#2151, CST), c-Myc (#1472-1, Epitomics), GAPDH (#GTX627408, GeneTex), Ki67 (#9449, CST), and LC3 (#12741, CST).

BAY-876 (i.e., N4-[1-(4-cyanobenzyl)-5-methyl-3-(trifluoromethyl)-1H-pyrazol-4-yl]-7-fluoroquinoline-2,4-dicarboxamide) was acquired from Medkoo Bioscience (Durham, NC, USA) (Cat No. 530485).

The ketogenic diet (Cat No. TP201450) was purchased from Trophic Animal Feed High-tech Co., Ltd. (Nantong, China).

### 2.3. Cell Lines and Mouse

The LS174T cell line (LS174T-TR4) was described in our previous studies [22]. The LS174T cells were subjected to blasticidin selection to confer resistance [33]. The LS174T cells were cultured in RPMI1640 medium (Sigma, R8758) supplemented with 5% Fetal Bovine Serum (Sigma F0926). The HCT116 cells were purchased from ATCC and were cultured in DMEM medium (Sigma, D6429) supplemented with 10% Fetal Bovine Serum (Sigma F0926). The HCT116 cell line was originally isolated from the colon of an adult male human with colon cancer. All the cells were incubated in a temperature-controlled incubator (NuAire, Plymouth, MN) set at 37 °C with 5% CO_2_.

The *RAG1*^−/−^ γc^−^ mice were kindly provided by Professor Min Fang and were generated as described [34,35]. The mice were housed under specific pathogen-free (SPF) conditions. The Recombination Activation Gene 1 (*RAG1*) plays a crucial role in V(D)J recombination, which occurs at seven unique loci, including loci in mature B and T lymphocytes. Mice with a deficiency in *RAG1* (*RAG1*^−/−^) are known as an immunodeficient strain due to the absence of functional B and T lymphocytes.

### 2.4. Western Blotting

Western blotting was conducted as previously reported [36]. The cells were seeded into 6-well plates and incubated for 24 h. Either dimethyl sulfoxide (DMSO) or compounds dissolved in DMSO at indicated concentrations were added to each well and incubated for an additional 24 h. The cells were lysed in 500 μL of lysis buffer containing 100 mmol/L NaCl, 50 mmol/L HEPES, 1% (*v*/*v*) glycerol, 2 mmol/L EDTA, 1 mmol/L Na_3_VO_4_, 50 mmol/L NaF, 1% (*v*/*v*) Triton X-100, and a protease inhibitor cocktail (Sigma, P8340). The cell lysates were centrifuged for 10 min (18,000× *g*), and the supernatants were mixed with 6 × protein loading buffer and boiled for 3 min. The standard Western blotting methods were carried out to analyze the samples using the appropriate antibodies. The original Western blot figures can be found in Supplementary Materials File S2.

### 2.5. Seahorse Assay for Oxygen Consumption Rate Measurement

The Seahorse assays were conducted following a standardized protocol [36]. The Seahorse XFe96 Extracellular Flow Analyzer (Agilent Technologies, Santa Clara, CA, USA) was used to measure the oxygen consumption. An XF96 Cell Culture microplate was used to culture the LS174T cells, with a seeding density of 2.5 × 104 cells in 80 μL medium. The plate was incubated overnight. On the next day, the cell culture media was replaced with Seahorse XF modified media. Oligomycin (1 μmol/L), FCCP (1 μmol/L), and a mixture of rotenone (1 μmol/L) and antimycin A (1 μmol/L) were sequentially added during the measurements. To identify whether the compounds were ETC complex V/ATP synthase inhibitors, an equal volume of DMSO or compounds dissolved in DMSO solution was used as a substitute for oligomycin. To assess the inhibitory effects of the compounds on ETC complex I or III, DMSO alone was used as a substitute for rotenone and antimycin A, or these compounds were dissolved in DMSO.

### 2.6. Measurements of Mitochondrial ETC Complex Activity Using PMP

The activity of the ETC complex was assessed utilizing the Agilent Seahorse Assay with an XF permeabilizer (PMP; Agilent), following the guidelines provided by the manufacturer. The mitochondrial assay solution (MAS) was prepared containing 70 mmol/L sucrose, 220 mmol/L mannitol, 5 mmol/L MgCl_2_, 10 mmol/L KH_2_PO_4_, 1 mmol/L EGTA, 2 mmol/L HEPES, and 0.2% (*w*/*v*) fatty acid-free BSA. Then, the cells were washed with MAS. Subsequently, the cell culture medium was changed with MAS containing 1 nmol/L PMP, 4 mmol/L adenosine diphosphate (ADP), and 10 mmol/L pyruvate. After the calibration process, the baseline OCR was determined by conducting a test that involved alternating cycles of mixing for 0.5 min, waiting for 0.5 min, and measuring for 2 min. After establishing the baseline definition, we proceeded with a sequential injection of DMSO, test compounds or rotenone (2 μmol/L), succinate (10 mmol/L), antimycin A (2 μmol/L), and a mixture of ascorbate (10 mmol/L) and N,N,N,N-tetramethyl-p-phenylenediamine (TMPD; 100 μmol/L). The data were subsequently standardized based on the density of the cells using Biotek Cytation 1 (BioTek Instruments, Winooski, VT, USA).

### 2.7. Cell Proliferation Inhibition Assay

A cell proliferation inhibition assay was carried out in accordance with a previously published protocol [37]. The cells were seeded at a density of 40,000 cells per well into 12-well plates and were cultured at 37 °C for 24 h. The cells were treated with 1 μL of the compounds that were dissolved in DMSO at a concentration of 10 mmol/L. DMSO was used as vehicle control. After 5 days, the medium was removed, and 200 μL of 0.25% trypsin was added. The cells were digested with trypsin and resuspended in phosphate-buffered saline (PBS) and counted using Vi-CELL XR 2.03 (Beckman Coulter, Inc., Brea, CA, USA). The relative growth rate, denoted as ratio R, represented the comparison between the number of viable cells in the compound treatment group and that in the DMSO treatment group, and the growth inhibition rate was calculated as (1 − R) × 100.

### 2.8. Calculation of Synergy Scores of BAY-876 and DBI-2

To evaluate the inhibitory effect of BAY-876, both alone and in combination with DBI-2 at predetermined concentrations, the cell proliferation inhibition assay was performed using 24-well plates on the LS174T and HCT116 cells. The concentrations of BAY-876 used for the final treatment were 0, 10, 30, 100, and 300 nmol/L in the presence or absence of DBI-2 at 0, 100, 300, 1000, and 3000 nmol/L in the LS174T cells. The concentrations of BAY-876 used for the final treatment were 0, 50, 100, 200, and 400 nmol/L and for DBI-2 were 0, 100, 200, 400, and 800 nmol/L in the HCT116 cells. The web application of SynergyFinder (version 2.0; http://www.synergyfinder.org accessed on 14 May 2022) was utilized to measure the synergy scores, with Bliss serving as the reference model and the default parameters being applied [38].

### 2.9. Quantification of Apoptotic Cells and Necrotic Cells by Flow Cytometry

The LS174T cells and HCT116 cells were cultured in 6-well plates at 37 °C for 24 h and treated with 3 μmol/L DBI-2 or 1 μmol/L BAY-876 for 24 h. The cells of the combination therapy group were treated with both DBI-2 and BAY-876. The cells were harvested and rinsed twice with cold PBS and resuspended in 500 μL Binding Buffer. Subsequently, the cells were stained with Annexin V-FITC and propidium iodide (PI) and incubated in the dark for 5 min. The percentages of apoptosis and necrosis were determined by flow cytometry (BeckmanCoulter, Brea, CA, USA).

### 2.10. MDC Staining

Using 24-well plates, 2 × 10^5^ LS174T cells and HCT116 cells were seeded into these plates and were cultured at 37 °C for 24 h. The cells were treated with 3 μmol/L DBI-2 or 1 μmol/L BAY-876 for 8 h. The combination therapy group was treated with both DBI-2 and BAY-876. The cells were stained with monodansylcadaverine (MDC; Solarbio, China) for 30 min at room temperature and washed with buffer three times. Fluorescence microscopy (the excitation filter wavelength was 355 nm and the blocking filter wavelength was 512 nm) was used to observe the autophagic vacuoles and to capture the images. 

### 2.11. Lactate Dehydrogenase (LDH) Enzymatic Activity Quantification in the Culture Medium

Using 12-well plates, 5 × 10^5^ LS174T cells and HCT116 cells were cultured in these plates at 37 °C for 24 h. The cells were treated with 3 μmol/L DBI-2 and 1 μmol/L BAY-876 alone or in combination for 24 h. The LDH concentrations of the LS174T cells and HCT116 cells were measured at 450 nm using 10 μL culture medium using the LDH activity detection kit (Solarbio, China) according to the manufacturer’s instructions. 

### 2.12. In Vivo Evaluation of Antitumor Effects in LS174T Xenografts

The LS174T cells were collected during the exponential growth phase and combined with an equivalent amount of Matrigel (Corning, Glendale, AZ, USA). These 2 × 10^6^ cells were injected subcutaneously into the lower flanks of each mouse. Receiving tumor cell injections, the mice were divided into control and treatment groups in a random manner once tumors had formed and reached an average volume of 100 mm^3^ (with each group consisting of three male and three female mice, with two tumors on each mouse).

After the establishment of tumors, DBI-2 was formulated in a mixture of ethanol (5%), 20% solutol and 20% Cremophor EL (50%) and citric acid (45%; 50 mmol/L, pH4.5) and the DBI-2 group received a daily intraperitoneal (IP) administration of DBI-2 at a dosage of 40 mg/kg mouse body weight. BAY-876 was dissolved in DMSO (10%), PEG400 (25%), Tween 80 (5%), and PBS (60%). The BAY-876 group received a daily dose of 3 mg/kg mouse body weight through gavage feeding (PO). The group receiving combination therapy underwent daily administration of DBI-2 at a dosage of 40 mg/kg in conjunction with BAY-876 administered at a dose of 3 mg/kg relative to the weight of the mice. All the mice of the control group, DBI-2 treatment group, BAY-876 treatment group, and combination therapy group received the standard diet ad libitum for the duration of the study. There were two groups that were fed the ketogenic diet (KD), and in addition to this, the KD-fed combined with DBI-2 treatment group was treated daily with DBI-2 at a dose of 40 mg/kg mouse body weight. The nutritional percent of the KD is as follows: 3.2% of Cal from carbohydrates, 8.9% of Cal from protein, and 74.2% of Cal from fat. The food was changed daily.

The measurements of the tumor sizes and mouse weights were conducted every three days, and the tumor volume (length × width^2^/2) was calculated. The experiment was terminated on day 12 and the mice were humanely sacrificed. The tumors of all the groups were isolated and fixed in 10% neutral buffered formalin before embedding in paraffin. The tumor sections were subjected to a histological examination using H&E and immunohistochemical staining. The plasma was collected and analyzed with the assay kit (Jiancheng Bioengineering Institute, Nanjing, China) according to the manufacturer’s protocol.

### 2.13. Hematoxylin and Eosin (H&E) Staining and Immunohistochemistry

The 6 μm sections were collected and dried for 2 h at 60 °C. The slides were deparaffinized and hydrated in xylene and graded ethanol solutions and washed two times in distilled water. For the H&E staining, the slides were placed in Hematoxylin (Biosharp) for 3 min and washed with tap water. Subsequently, differentiation was carried out in 1% hydrochloride in 70% ethanol for 30 s. After differentiation, the slides were rinsed in tap water and incubated in Eosin solution (Biosharp) for 5 min. Finally, the slides were dehydrated in a graded series of ethanol and xylene. 

For immunohistochemistry, the slides were heated in a microwave submersed in 0.01 mol/L citrate for antigen unmasking. After these slides were subjected to a 10 min incubation in a solution containing 3% hydrogen peroxide, the slides underwent a blocking process for 15 min at 37 °C and were treated with Ki-67, p-AMPK, and AMPK primary antibody overnight at 4 °C. The next day, secondary antibodies were added to the slides for 15 min at 37 °C. A DAB substrate reaction was completed followed by a Streptavidin–Biotin reaction. Hematoxylin was used for staining the nucleus. 

### 2.14. Statistics

The major biological assays were repeated at least twice. For the animal studies, a total of six mice were included in each group with two tumors on the lower flanks of every individual mouse. The data were expressed as the mean ± SEM and a statistical analysis was performed with GraphPad Prism 5. A one-way ANOVA followed by Tukey’s multiple comparisons test were used to calculate the statistical significance among the groups, and *p* < 0.05 was defined as statistically significant. 

## 3. Results

### 3.1. Identification of DBI-2 as a Novel AMPK Activator

**Figure 1:**
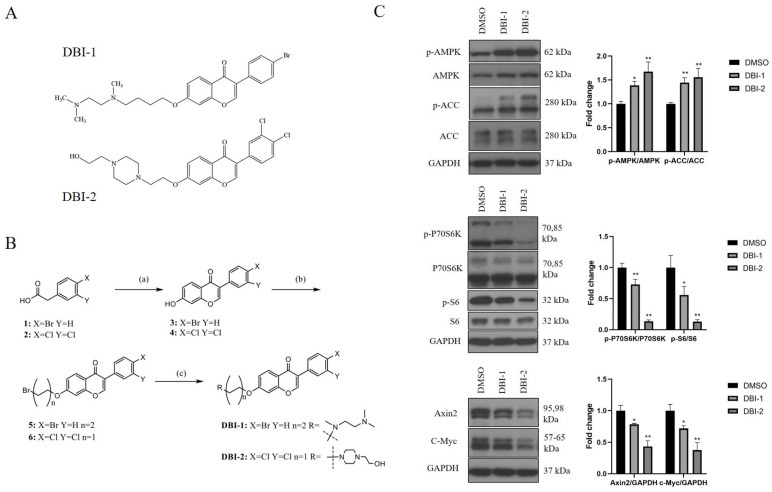
Small-molecule AMPK activators, DBI-1, and DBI-2. (**A**) Structures of DBI-1 and DBI-2. (**B**) Synthesis of DBIs. Reagents and conditions: (a) resorcinol, DMF, PCl_5_, BF_3_·Et_2_O, 85 °C, 4 h; (b) appropriate dibromoalkane, K_2_CO_3_, DMF, 80 °C, 3 h; and (c) amine, DIPEA, NaI, DMF, 60 °C, 4 h. (**C**) DBI-1 and DBI-2 (3 μmol/L) increased AMPK phosphorylation (p-AMPK) and ACC phosphorylation (p-ACC), indicators of AMPK activation, and decreased the P70S6K phosphorylation (p-P70S6K) and S6 phosphorylation (p-S6), indicators of the mTOR signaling, and reduced the expression of Axin2 and c-Myc, key markers for the Wnt signaling, in LS174T CRC cells after 24 h treatment. Data are presented as the means ± SEM. * *p* < 0.05, and ** *p* < 0.01 versus control group, n = 3. One-way ANOVA followed by Tukey’s multiple comparisons test were applied as the statistical method.

In our prior research [22], we identified a diaminobutoxy-substituted isoflavonoid (DBI-1) as a mitochondrial complex I inhibitor (Figure 1A). DBI-1 reduced the ETC-dependent energy production and activated the AMP-activated protein kinase (AMPK), a crucial energy sensor in the cells. Using an AMPK phosphorylation assay as a readout, we synthesized and analyzed a panel of new DBI-1 analogs, and we identified DBI-2 (Figure 1A,B) as a promising agent that significantly induced AMPK phosphorylation (activation) and ACC (Acetyl-CoA Carboxylase, a downstream target of AMPK) phosphorylation (inhibition) at 3 μmol/L in the LS174T CRC cells (Figure 1C). We reported previously that reducing ATP production by OXPHOS inhibitors or mitochondrial uncouplers not only activated the AMPK signaling pathway but also inhibited the mTOR and Wnt signaling pathways in CRC cells [22,36,39]. As expected, the signaling pathway of mTOR (p70S6K and pS6) was suppressed by DBI-2, and the expression of Axin2 and c-Myc, the major target genes of Wnt signaling, was also inhibited. DBI-2 was more active than DBI-1 in these assays (Figure 1C). These results demonstrated that DBI-2 induced growth suppression by activating AMPK and inhibiting the mTOR and Wnt pathways.

### 3.2. Combinational Effects of DBI-2 and BAY-876 on Cell Signaling Pathways in CRC Cells

**Figure 2:**
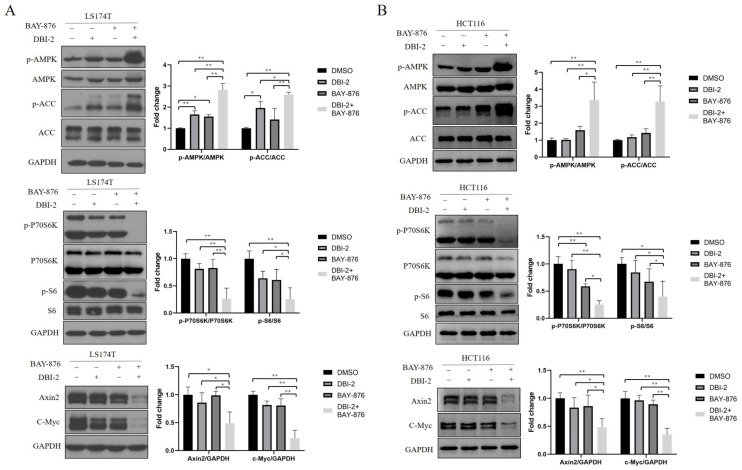
The impact of DBI-2 and BAY-876 on cell signaling pathways in LS174T (**A**) and HCT116 (**B**) CRC cells. Combination of DBI-2 (3 μmol/L) and GLUT1 inhibitor BAY-876 (1 μmol/L) resulted in enhanced depletion of Axin2 and c-Myc, increase in p-AMPK and p-ACC, and depletion of p-P70S6K and p-S6. Data are presented as the means ± SEM. * *p* < 0.05, and ** *p* < 0.01, n = 3. One-way ANOVA followed by Tukey’s multiple comparisons test were applied as the statistical method.

We reported that complex I inhibitors, including rotenone, metformin, and DBI-1, had synergistic effects with BAY-876, a GLUT1 inhibitor, on CRC cell growth inhibition [22]. DBI-2 alone at 3 μmol/L activated AMPK and inhibited the mTOR and Wnt signaling pathways in the CRC cells (Figure 1C). To test the combinatorial effects of DBI-2 and BAY-876, we treated the LS174T and HCT116 CRC cells with either 3 μmol/L DBI-2 or 1 μmol/L BAY-876 as well as with the combination of these two compounds. The combination of DBI-2 and BAY-876 was significantly more effective than either DBI-2 or BAY-876 alone in activating AMPK and inhibiting the mTOR and Wnt signaling pathways (Figure 2). 

### 3.3. DBI-2 and GLUT1 Inhibitor, BAY-876, Synergistically Inhibited CRC Cells Proliferation In Vitro

**Figure 3:**
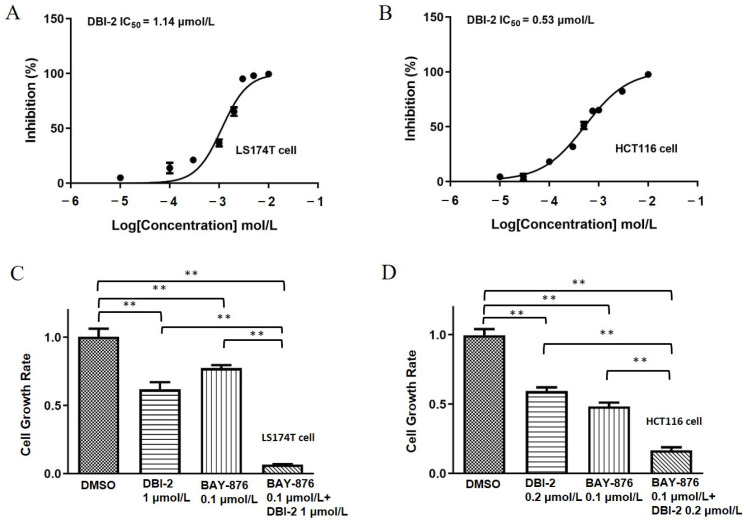
DBI-2 and BAY-876 synergically inhibited CRC cells in vitro. (**A**,**B**) DBI-2 inhibited colorectal cancer cell LS174T (**A**) and HCT116 (**B**) proliferation. The IC_50_ of DBI-2 to inhibit the proliferation of LS174T cells and HCT116 cells was 1.14 μmol/L and 0.53 μmol/L, respectively. (**C**,**D**) Combinatorial effects of DBI-2 and BAY-876 on CRC cell proliferation. The cells were exposed to DBI-2 or BAY-876 at specified concentrations for a duration of 5 days, and the number of viable cells was determined. Data are presented as the means ± SEM. ** *p* < 0.01, n = 3. One-way ANOVA followed by Tukey’s multiple comparisons test were applied as the statistical method.

DBI-2 repressed the proliferation of the LS174T and HCT116 CRC cells with an IC_50_ of 1.14 and 0.53 μmol/L, respectively (Figure 3A,B). BAY-876 repressed the proliferation of the LS174T and HCT116 CRC cells with an IC_50_ of 0.1–0.2 μmol/L [22]. The co-administration of BAY-876 and DBI-2 dramatically repressed the cell proliferation in the LS174T and HCT116 cell lines (Figure 3C,D).

**Figure 4:**
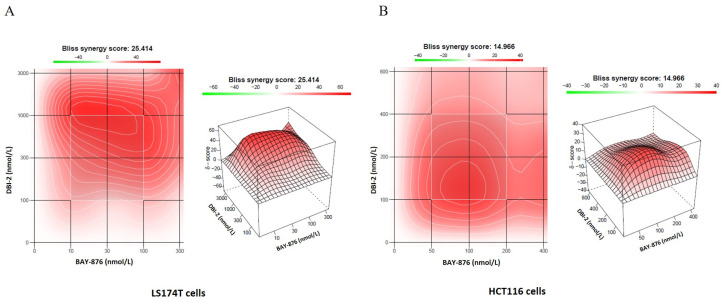
Synergistic effects of DBI-2 and BAY-876 on LS174T and HCT116 CRC cells. (**A**,**B**) DBI-2 and BAY-876 synergistically inhibited the proliferation of colorectal cancer cells. The cells were exposed to DBI-2 and BAY-876 at specified concentrations over a period of 5 days, and the number of viable cells was determined. Synergy scores were measured utilizing SynergyFinder, with Bliss serving as the benchmark model. The synergy scores for drug combinations of DBI-2 and BAY-876 were 25 for LS174T cells (**A**) and 15 for HCT116 cells (**B**).

To verify that the combinational effects of BAY-876 and DBI-2 were synergistic rather than additive, we analyzed the Bliss synergy scores of the BAY-876/DBI-2 combination using the SynergyFinder program (version 2.0) [38]. For the LS174T cells, the synergy score for the combination of DBI-2 (0.1–3 μmol/L) and BAY-876 (10–300 nmol/L) was 25. For the HCT116 cells, the synergy score was 15 for the combination of DBI-2 (100–800 nmol/L) and BAY-876 (50–400 nmol/L). These data suggested a notable synergistic effect because the synergy score values exceeded the cut-off value (i.e., 10) associated with no synergistic effects by a significant margin (Figure 4A,B).

**Figure 5:**
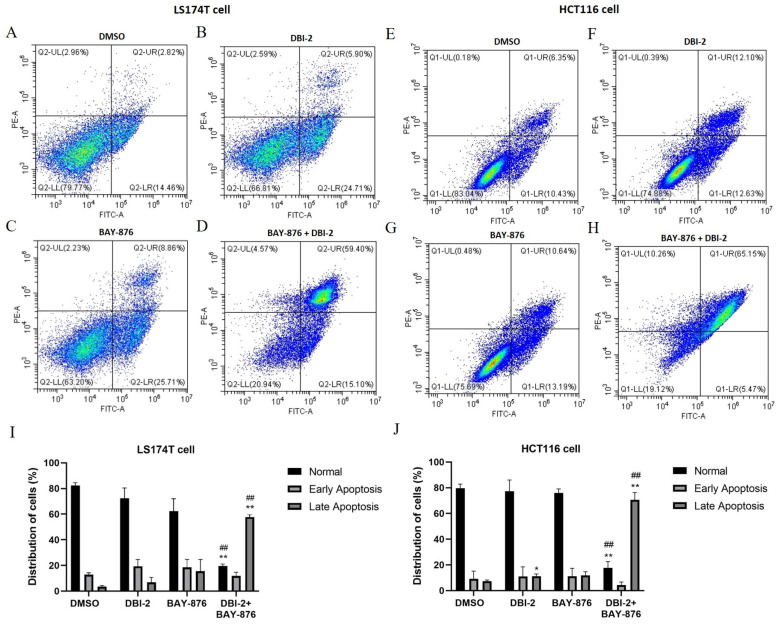
Synergistic effects of DBI-2 and BAY-876 on inducing apoptosis in LS174T cells and HCT116 cells through flow cytometry analysis using Annexin-V-FITC and propidium iodide (PI) dual staining. (**A**,**E**) Cells were treated with DMSO. The apoptosis rate was 16% and 17%, respectively. (**B**,**F**) Cells were treated with DBI-2 at 3 μmol/L. The percentage of apoptotic cells was 26% and 22%, respectively. (**C**,**G**) Cells were treated with BAY-876 at 1 μmol/L. The apoptosis rate was 34% and 23%, respectively. (**D**,**H**) Cells were treated with the combination of DBI-2 (3 μmol/L) with BAY-876 (1 μmol/L). The apoptosis rate was 70% and 75%, respectively. (**I**,**J**) Percentage of normal, early apoptotic, and late apoptotic cells in LS174T cells and HCT116 cells. Data are presented as the means ± SEM. * *p* < 0.05, and ** *p* < 0.01 versus control group, ^##^ *p* < 0.01 versus DBI-2 or BAY-876 group, n = 3. One-way ANOVA followed by Tukey’s multiple comparisons test were applied as the statistical method.

We also analyzed the combinational effects of DBI-2 and BAY-876 on CRC cell apoptosis. Annexin V-FITC was used to quantify the externalization of phosphatidylserine, while the dye, propidium iodide (PI), was used to evaluate the loss of membrane integrity as a marker of apoptosis and necrosis, respectively [40]. After 24 h treatment, flow cytometry was used to measure the cells for Annexin V FITC and PI dual labeling. For the LS174T cells and HCT116 cells, the apoptosis rate of the DMSO-treated group was 16% and 17%, respectively (Figure 5A,E). The percentage of apoptotic cells of the DBI-2 (3 μmol/L)-treated group was 26% and 22%, and that of the BAY-876 (1 μmol/L)-treated group was 34% and 23%, respectively (Figure 5B,C,F,G). The combination of DBI-2 with BAY-876 resulted in a significant increase in the apoptosis rate of 70% and 75%, respectively (Figure 5D,H,I,J), relative to the treatments with DBI-2 or BAY-876 alone.

**Figure 6:**
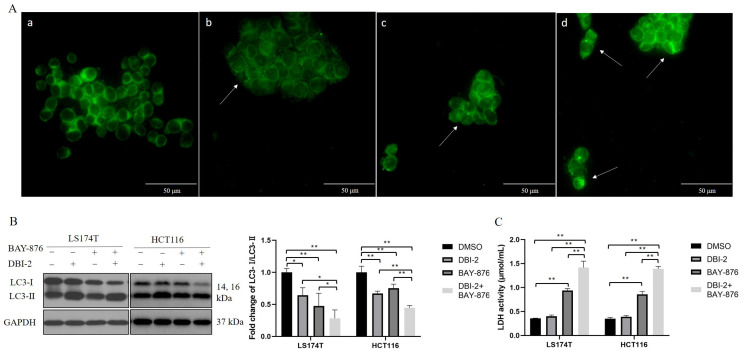
Synergistic effects of DBI-2 and BAY-876 on inducing autophagy and necrosis in LS174T cells and HCT116 cells. (**A**) Detection of autophagic vacuoles by MDC staining in LS174T cells treated with DBI-2 and BAY-876 alone and in combination for 8 h (a: DMSO; b: DBI-2; c: BAY-876; and d: DBI-2 and BAY-876). (**B**) Expression of LC3-Ⅰ and LC3-Ⅱ in LS174T cells and HCT116 cells after 8 h treatment. (**C**) The LDH activity in the culture medium of LS174T cells and HCT116 cells. Data are presented as the means ± SEM. * *p* < 0.05, and ** *p* < 0.01, n = 3. One-way ANOVA followed by Tukey’s multiple comparisons test were applied as the statistical method.

After studying the apoptosis and necrosis levels in the CRC cells in response to DBI-2, BAY-876, or their combination (Figure 5), we next investigated if these compounds induced cell death via autophagy. The LS174T cells were stained with MDC, a fluorometric probe known as a specific marker for autophagic organelles. The MDC staining demonstrated that the drug-treated groups exhibited more MDC-labeled autophagic vacuoles compared to the control group (Figure 6A). Furthermore, we evaluated the conversion of cytosolic microtubule-associated protein I light chain 3 (LC3) to autophagosome-associated LC3-II, a widely recognized indicator of autophagosome formation. The conversion of LC3-I to LC3-II was remarkably increased in the drug-treated LS174T cells and HCT116 cells, an outcome indicating that DBI-2 and BAY-876 induced autophagy in the CRC cells (Figure 6B). To corroborate necrosis, we analyzed the LDH enzymatic activity in the culture medium. The LDH activity was significantly increased in the BAY-876 treatment group and the combination treatment group, an outcome indicating that BAY-876 exerted a prominent influence on cell necrosis. In summary, these results demonstrated that DBI-2 and BAY-876 induced apoptosis, necrosis, and autophagy.

### 3.4. DBI-2 Inhibited Mitochondrial Complex I

**Figure 7:**
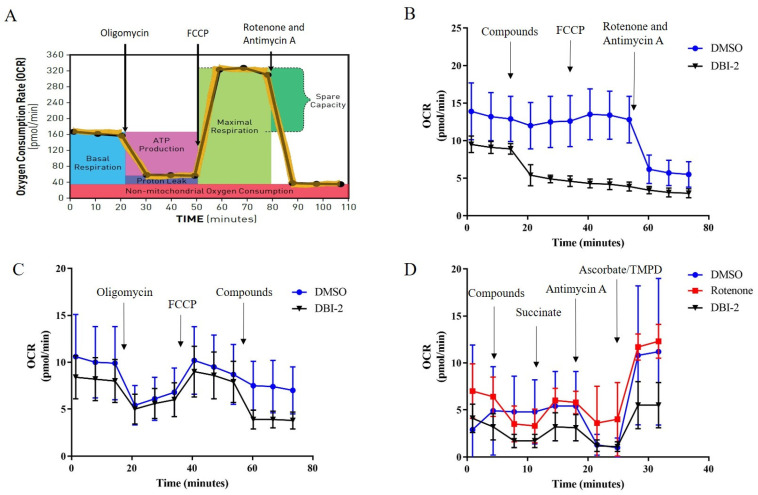
DBI-2 inhibited mitochondrial complex I. (**A**) Model of standard Seahorse OCR assay. Oligomycin suppressed ATP-linked respiration and reduced OCR. FCCP rescued oligomycin-inhibited OCR and rotenone and antimycin A inhibited the FCCP-induced OCR. (**B**) Oligomycin was replaced with DBI-2, the OCR was inhibited, but the decreased OCR could not be rescued by the addition of FCCP, a finding that indicated that DBI-2 does not function as a mitochondrial uncoupler or an inhibitor of complex V/ATP synthase. (**C**) Substituting rotenone/antimycin A with DBI-2 resulted in a reduction in the OCR, a finding that indicated that DBI-2 exerted inhibitory effects on either mitochondrial complex I or complex III. (**D**) Succinate, the substrate for complex II, could rescue the inhibited OCR caused by rotenone or DBI-2, indicating that DBI-2 is a complex I inhibitor.

We previously established that DBI-1 was a mitochondrial complex I inhibitor. To determine if DBI-2 also played a similar role in regulating mitochondrial function, we performed Seahorse assays to investigate the effect of DBI-2 on oxygen consumption rates (OCRs). In the standard assay, oligomycin, a known inhibitor of mitochondrial complex V/ATP synthase, decreased the OCR. A mitochondrial uncoupler, FCCP, rescued the oligomycin-inhibited OCR. A mixture of the complex I inhibitor, rotenone, and complex III inhibitor, antimycin A, inhibited the FCCP-induced OCR (Figure 7A). We replaced the oligomycin with DBI-2 in the standard assay. The OCR was inhibited, but the FCCP could not rescue the decreased OCR (Figure 7B). This result indicated that DBI-2 does not possess the ability to uncouple mitochondria or inhibit complex V/ATP synthase. We subsequently substituted rotenone/antimycin A with DBI-2 and observed a comparable inhibition of the OCR by DBI-2 (Figure 7C). This finding suggested that DBI-2 inhibited the activity of complex I and/or complex III of the ETC.

To delineate whether DBI-2 inhibited complex I or complex III, we assessed the substrate-specific OXPHOS activity by utilizing plasma membrane permeabilizer (PMP)-treated CRC cells (Figure 7D). In these studies, the plasma membrane permeabilizer, a recombinant mutant of cholesterol-dependent cytolysin derived from *Clostridium perfringens*, underwent oligomerization to create pores that specifically penetrated the cytoplasmic membrane. As a result, the permeabilizer facilitated the transportation of solutes and large proteins weighing >200 kDa [41,42,43]. Thus, the cells were permeabilized and treated with pyruvate, a complex I-linked substrate. A mixture of DMSO with rotenone, the complex I inhibitor, or with DBI-2 was subsequently added. DBI-2 reduced the respiration linked to the pyruvate/NADH. This result was comparable to that of the complex I inhibitor rotenone. Next, succinate was added to facilitate the respiration of succinate dehydrogenase (complex II) that bypassed complex I. The respiration increased in both the rotenone-treated and DBI-2-treated permeabilized cells, a fact that excluded the possibility that DBI-2 was an inhibitor of complexes II, III, and IV. Finally, antimycin A, a complex III inhibitor, was added to the system and the OCR was again suppressed. However, this suppression was alleviated by the injection of TMPD as an electron donor for complex III, which bypassed the complex III blockade and directly supplied electrons to cytochrome c oxidase (complex IV). In summary, the Seahorse OCR assay data utilizing specific inhibitors targeting the ETC complex and PMP-permeabilized cells confirmed that DBI-2 was a complex I inhibitor that disrupted mitochondrial function.

### 3.5. Ketogenic Diet Enhanced the Therapeutic Efficacy of DBI-2 on CRC Xenograft Models

**Figure 8:**
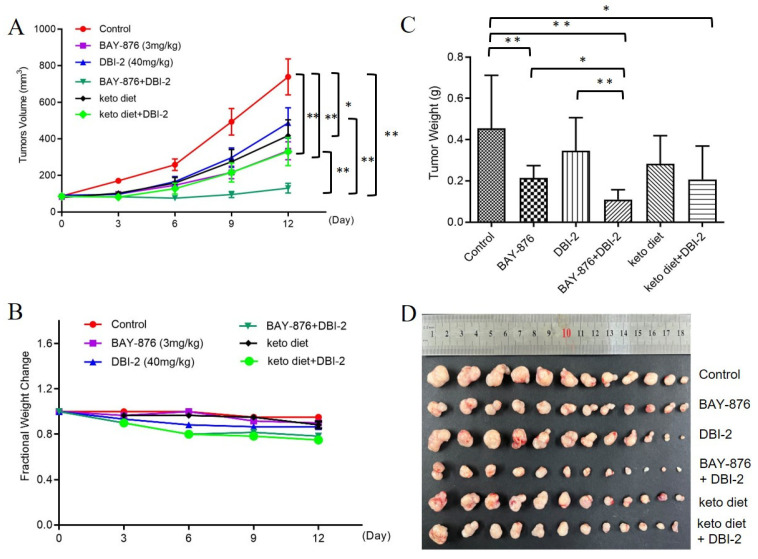

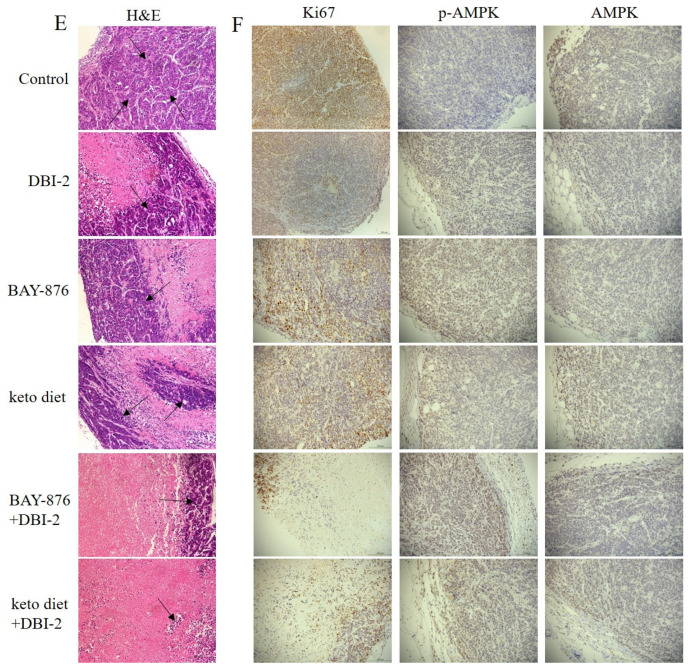
The combination of DBI-2/KD and the GLUT1 inhibitor BAY-876 exhibited a synergistic inhibitory effect on colon cancer cell xenografts in vivo. (**A**–**C**) The combination of DBI-2 (40 mg/kg/day, IP) with BAY-876 (3 mg/kg/day, PO) or ketogenic diet feeding significantly inhibited tumor growth in *RAG1*^−/−^ *γc*^−^ mice. Notably, this treatment regimen did not induce any noticeable adverse effects based on body weight. (**D**) Pictures of tumors after treatment for 12 days. (**E**) H&E staining of tumor sections (arrows). (**F**) IHC staining of Ki67, p-AMPK, and AMPK. Data are presented as the means ± SEM. * *p* < 0.05, and ** *p* < 0.01, n = 12. One-way ANOVA followed by Tukey’s multiple comparisons test were applied as the statistical method.

In the cell-based assays, DBI-2 and BAY-876 had synergistic effects on CRC cell inhibition in which they blocked both the glycolytic pathway and OXPHOS. However, BAY-876 has a rather narrow therapeutic window and is not an FDA-approved drug [21]. Consequently, we hypothesized that a ketogenic diet (KD) would reduce carbohydrate uptake and thus mimic the effects of GLUT1 inhibitors. To test this hypothesis, we explored the in vivo therapeutic effects of DBI-2 and its combination with a KD in *RAG1*^−/−^ *γc*^−^ mouse models with BAY-876 as a control. A CRC xenograft model was established using LS174T cells in *RAG1*^−/−^ *γc*^−^ mice. The tumor growth was inhibited by daily intraperitoneal (IP) administration of 40 mg/kg/day of DBI-2 or oral administration of 3 mg/kg of BAY-876 or daily ketogenic diet feeding for 12 days (Figure 8A). The co-administration of BAY-876 (3 mg/kg/day, PO) and DBI-2 (40 mg/kg/day, IP) resulted in a more pronounced suppression of tumor growth than that seen using the individual compounds (Figure 8A). IP injection of DBI-2 at 40 mg/kg/day together with ketogenic diet feeding also significantly inhibited tumor growth, but the effect was inferior to the combination of DBI-2 and BAY-876 (Figure 8A). All the treatment groups exhibited negligible toxicity in terms of the effects on the growth rate of the mice body weight (Figure 8B). After a duration of 12 therapy days, the tumors were dissected and weighed, and the tumor slides were analyzed by H&E and IHC staining (Figure 8C–F). The immunohistochemical results showed a notable decrease in the levels of Ki-67 expression, an outcome that provided an indicator for cellular proliferation in the tumor samples treated with the combined administration of DBI-2 and BAY-876 and DBI-2 in combination with ketogenic diet feeding. The levels of p-AMPK and AMPK expression were increased consistently with those of the in vitro experiments (Figure 8F). In conclusion, DBI-2 has a more pronounced anticancer effect than the effects seen when DBI-2 was utilized individually or in combination with other inhibitors targeting energy metabolism, such as inhibitors of glucose transport. The drug/diet combination provided a potential therapeutic strategy for cancer treatment.

### 3.6. Combination of DBI-2 with BAY-876 Reduced Blood Glucose, Plasma TG, T-CHO, and LDL-C

**Figure 9:**
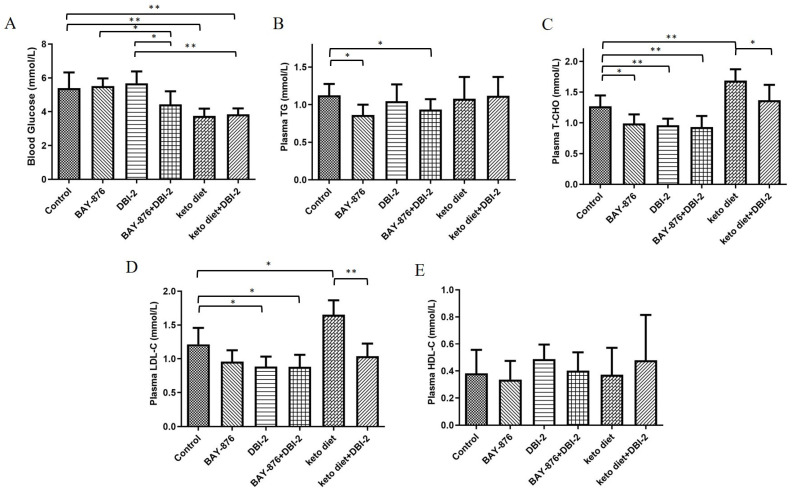
Combination of DBI-2 with BAY-876 reduced blood glucose, plasma triglycerides (TG), T-CHO, and LDL-C. (**A**) Blood glucose tested from *RAG1*^−/−^ *γc*^−^ mice tails. (**B**–**E**) The plasma TG, T-CHO, LDL-C, and HDL-C tested in tumor-bearing animals. Blood obtained via retro orbital eye bleeds prior to sacrifice. Blood samples spun and plasma separated. Data are presented as the means ± SEM. * *p* < 0.05, and ** *p* < 0.01, n = 6. One-way ANOVA followed by Tukey’s multiple comparisons test were applied as the statistical method.

The three treatments, namely, [A] the combination of DBI-2 and BAY-876; [B] the use of a KD diet; and [C] the combination of DBI-2 and a KD diet, showed significant reductions in the blood glucose (Figure 9A). It has been shown that the synergistic effect of nutrient restriction and high fat affects the metabolism and liver pathology in mice [44]. Because high cholesterol levels are associated with high-fat diets, analyses of triglycerides, cholesterol, LDL-C, and HDL-C were performed [45]. Consistent with these results, the mice fed the ketogenic diet showed a notable rise in the cholesterol and LDL-C levels (Figure 9C,D). DBI-2 combined with BAY-876 significantly reduced the triglyceride, cholesterol, and LDL-C levels, an outcome that was better than a ketogenic diet combined with DBI-2 (Figure 9B–D). There were no significant changes in the HDL-C level among all the groups. Taken together, these results indicated that DBI-2 in combination with BAY-876 is relatively healthy and nutritionally safe.

## 4. Discussion

Colorectal cancer is a common malignant cancer that displays heterogeneity in genetics and metabolism. Genetic factors play an important role in the occurrence of colorectal cancer, as many gene mutations are associated with increased risk, including mutations in APC, KRAS, TP53, and other genes. These mutations disrupt key processes such as cell proliferation, apoptosis, and DNA repair, all of which promote the formation of cancer cells [46,47]. In addition, metabolic abnormalities are also considered as an important aspect of heterogeneity in colorectal cancer. Metabolic pathways participate in energy production and substance synthesis within cells and may become imbalanced in colorectal cancer. Impaired glucose metabolism and mitochondrial respiration may lead to insufficient energy production and affect normal cellular functions. The development of therapeutic strategies should consider the heterogeneous characteristics of colorectal cancer. For example, targeted drugs or treatments would be selected based on whether patients have certain mutations or metabolic abnormalities.

The PI3K/AKT signaling pathway is crucial in regulating glucose metabolism, as it facilitates glycolysis and inhibits gluconeogenesis. This pathway directly impacts glucose metabolism by phosphorylating various metabolic enzymes or regulators of nutrient transport [48]. The tumor suppressor PTEN exerts its function by negatively regulating the oncogenic PI3K/AKT pathway [49]. The effects of MYC on glucose metabolism are mediated through the upregulation of key components, such as the glucose transporter GLUT1 and glycolytic enzymes, including hexokinase 2 (HK2), phosphofructokinase-M 1 (PFKM1), and enolase 1 (ENO1) [50,51]. When glucose is abundant, mutant p53 enhances glycolysis by facilitating the relocation of GLUT1 to the cells’ outer membranes. This process aids in generating energy and supplying essential components for cancer development [52]. Targeting genetic defects or metabolic abnormalities is thus an important strategy for colorectal cancer therapy.

We have a long-standing interest in developing natural products and their semisynthetic derivatives for cancer treatment [22,36,37,39,53,54,55,56,57,58]. We have developed diaminobutoxy-substituted isoflavonoid (DBI) compounds that activated AMPK and inhibited the proliferation of CRC cells by targeting mitochondrial complex I (Figure 1). DBI-2 emerged from SAR studies as a new DBI analog with promising properties. DBI-2 had synergistic effects with BAY-876, a GLUT1 inhibitor, on CRC cells in vitro and in vivo. In addition, a ketogenic diet also enhanced the therapeutic efficacy of DBI-2 in CRC xenograft mouse models (Figure 8). Although our long-term goal is to conduct clinical trials to evaluate these novel metabolic inhibitors, we are still working on the optimization of DBIs to improve their in vivo efficacy and to reduce their potential toxic effects. We are also trying to determine if any genetic or metabolic alterations in cancer cells affect the efficacy of these compounds. This information will be important for patient selection in future clinical trials.

Mechanistically, DBI-2 inhibited mitochondrial complex I activity (Figure 7), disrupted the OXPHOS process, and reduced ATP generation in mitochondria. The decrease in ATP production led to an increase in the AMK:ATP ratio and, consequently, led to the activation of AMPK. The mitochondrial complex I serves as an initial component of the electron transport chain (ETC) that facilitates the oxidation of NADH derived from the tricarboxylic acid (TCA) cycle and fatty acid β-oxidation pathway and that interdicts the essential transfer of electrons and protons required for ATP synthesis [59,60]. Targeting mitochondrial complex I of the mitochondrial electron transport chain in cancer affects the macromolecular synthesis and energy production and has emerged as a compelling therapeutic strategy in recent years. BAY-876, a well-known GLUT1 inhibitor, reduced glucose uptake and inhibited glycolysis. Cancer cells, exposed to BAY-876, are dependent on alternative energy sources, such as fatty acids and amino acids, that enter TCA and OXPHOS to produce ATP. Therefore, the simultaneous blocking of the two main energy supply pathways favored by cancer cells synergistically decreased the growth of cancer cells (Figure 2, Figure 3, Figure 4, Figure 5 and Figure 6). This dual inhibition would result in a particularly effective inhibition of cancer cell growth and might overcome therapeutic resistance through metabolic plasticity [61,62]. However, it should be pointed out that there are still some challenges and unknown factors before further promoting this combination therapy to clinical practice. For example, how to determine appropriate dosages, select patients, and evaluate potential toxic side effects all need to be explored and solved deeply. 

The solid tumors consist of multiple cell types derived from diverse origins that engage in intercellular communication to establish a dynamic and intricate network that governs the progression of tumor formation. The GLUT1 inhibitor, BAY-876, might inhibit not only colon cancer cells but also other cells of the tumor microenvironment, like T cells, immune cells, cancer-associated fibroblasts, and endothelial cells. The metabolic shift to glycolysis is ascribed to hypoxic conditions and the activation of the hypoxia-inducible factor (HIF). The central region of a solid tumor is characterized by areas of hypoxia, where there is insufficient oxygen and nutrients available. Many HIF1 target genes, including GLUT1 (SLC2A1) HK2, GPI, ENO1, and LDHA, were involved in glucose metabolism. The effects of GLUT1 and complex I inhibitors on these genes should be investigated in future studies.

BAY-876 was the first orally available and highly specific GLUT1 inhibitor. In a mouse model of an ovarian cancer xenograft, the effective dose of BAY-876 (4.5 mg/kg/day) also induced significant weight loss, indicating that BAY-876 had an unfortunate, narrow therapeutic window [21]. Absent GLUT1 inhibitors approved for human use, we found that the combination treatment of a KD diet and DBI-2 significantly inhibited tumor growth, although admittedly the effect was less than that of the DBI-2 and BAY-876 combination. We hypothesized that the combination of a ketogenic diet and DBI-2 inhibited two important energy-producing pathways of cancer cells, namely, glycolysis and OXPHOS, and this inhibition resulted in a dramatic effect on tumor growth. A ketogenic diet reduced the glucose metabolism of cancer cells and enabled the body to utilize its fat stores as a primary source of energy, facilitating accelerated fat burning. The KD group and the combined DBI-2 and KD treatment group showed a notable reduction in blood glucose levels (Figure 9A) consistent with the results reported by Poff et al. [63]. The combination of DBI-2 and BAY-876 showed a considerable reduction in triglycerides, cholesterol, and LDL-C. The DBI-2 and KD treatment group was able to alleviate the high levels of cholesterol and LDL-C caused by KD feeding alone. These results indicate that the combination of DBI-2 and BAY-876 is nutritionally safe and effective in inhibiting tumor growth. 

BAY-876 represented the first compound to demonstrate strong selectivity toward GLUT isoforms, specifically targeting GLUT-1 [20]. Ma et al. demonstrated the potent inhibition of ovarian cancer growth in OVCAR3 cells with BAY-876, achieving an IC_50_ value of up to 60 nM [64]. However, the treatment with BAY-876 showed resistance in the A2780 ovarian cancer cell line [21]. The polyphenol compound WZB117, also a GLUT1 inhibitor, effectively suppressed the growth and viability of A549 cells with IC_50_ values between 10 and 30 µM. In contrast, the non-malignant cell line NL20 (lung epithelium) exhibited resistance [21]. The utilization of xenograft models (A549) demonstrated a 70% reduction in tumor volume after 10 weeks of daily intraperitoneal injection (10 mg/kg), resulting in a loss of approximately 1 to 2 g of body weight compared to untreated control mice [21]. These results indicated that the optimization of GLUT1 inhibitors could increase the in vivo efficacy and reduce the potential toxicity of these inhibitors. 

It has been reported that a ketogenic diet has been used to treat a variety of cancers, including glioma [65], prostate cancer [66], lung cancer [67], gastric cancer [68], and head and neck cancers (HNCs) [69]. The ketogenic diet has the potential to assist individuals diagnosed with Type 2 diabetes in achieving weight loss goals and effectively controlling their blood sugar levels. The ketogenic diet may reduce the risk of developing cardiovascular disease by effectively lowering blood pressure, enhancing HDL cholesterol levels, and decreasing triglyceride levels. The ketogenic diet offers numerous advantages, although it may be accompanied by certain adverse effects. One of the indications of entering ketosis might manifest as “keto flu”, characterized by symptoms such as gastrointestinal discomfort, cephalalgia, constipation, and fatigue. A model of cancer cachexia demonstrated a delay in tumor growth while also exhibiting an accelerated onset of cachexia and shortened survival in mice fed a ketogenic diet [70]. The potential of the KD as an adjuvant therapy for certain tumors in the future is promising; however, extensive clinical studies are still required to substantiate this claim.

In vitro, the inhibitory effect of 1 μmol/L DBI-2 on the LS174T cell was 49%. Meanwhile, in the xenograft model, DBI-2 (40 mg/kg/day, IP) exhibited a tumor suppression rate of 34% after a treatment duration of 12 days. These findings indicate that the in vitro efficacy of DBI-2 surpasses the in vivo efficacy. The growth rate of the mice body weight on the CRC xenograft model was negligibly affected by toxicity in all the treatment groups. Further studies are needed to investigate the potential toxicity of higher doses of DBI-2. The possibility exists that cancer cells may possess the capacity to develop resistance against DBI-2. The potential strategy of synergistically combining DBI-2 with other energy metabolism inhibitors can be explored to overcome this resistance. In addition to CRC, we also performed the proliferation inhibition assay on a breast cancer cell (OVC-8) and prostate cancer cell (PC3). The inhibitory effect of DBI-2 on an OVC-8 cell reached 96% at 1 μmol/L. The inhibitory effect of 0.3 μmol/L DBI-2 on a PC3 cell was observed to be 62%, suggesting the potential therapeutic efficacy of DBI-2 in multiple cancer types. 

These findings, taken together, suggest that the further exploration of combination therapy targeting energy metabolism using a combination of OXPHOS inhibitors and the KD or glycolytic inhibitors may lead to new approaches for cancer treatment.

## 5. Conclusions

In conclusion, we identified DBI-2 that activated AMPK and inhibited the proliferation of CRC cells by targeting mitochondrial complex I. In vitro and in vivo experiments demonstrated that the combination of DBI-2 and BAY-876, a GLUT1 inhibitor, exhibited synergistic effects on CRC cells. Furthermore, the therapeutic effectiveness of DBI-2 in CRC xenograft mouse models was enhanced by implementing a ketogenic diet in combination with DBI-2 treatment. These results indicated that investigating the potential combination of special diets and metabolic modulators may lead to effective future strategies for cancer treatment.

## 6. Patents

A PCT patent (PCT/US22/052038) has been filed by the University of Kentucky Research Foundation and Hebei Normal University in accord with university policies.

## Data Availability

The authors generated the data and incorporated it into the article.

## Supplementary Materials

The following supporting information can be downloaded at: https://www.mdpi.com/article/10.3390/cancers16071399/s1, Supplementary File S1: Chemical synthesis and characterization; Supplementary File S2: Original Western blot figures; Figure S1: DBI-2 and BAY-876 synergically inhibited CRC cells in vitro.
