# Supplementary material for: Inhibition of Carbohydrate Metabolism Potentiated by the Therapeutic Effects of Oxidative Phosphorylation Inhibitors in Colon Cancer Cells
Source: Cancers (Basel). 2024 Apr 2;16(7):1399. doi: 10.3390/cancers16071399 (PMC11010912; doi:10.3390/cancers16071399)
Supplement: Supplementary file 1 [file cancers-16-01399-s001.zip › cancers-2876730-Figure S2.pdf]

Supplementary File S2: Representative original western blot figures presented in Figure 1C and Figure2.

Figure 1C.

p-ACC
280 kDa

p-AMPK
62 kDa

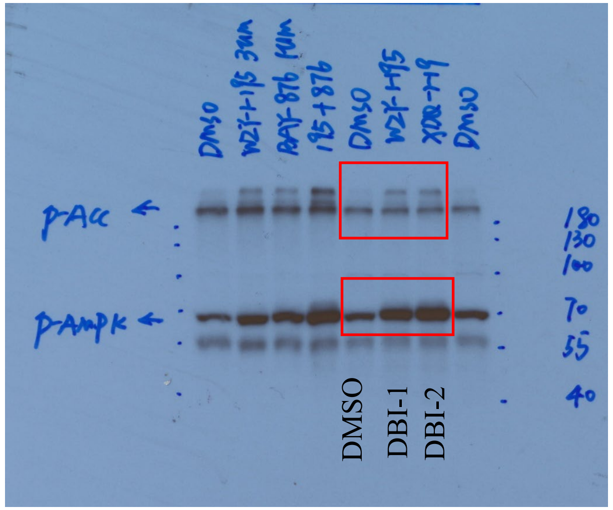

ACC
280 kDa

AMPK
62 kDa

GAPDH
37 kDa

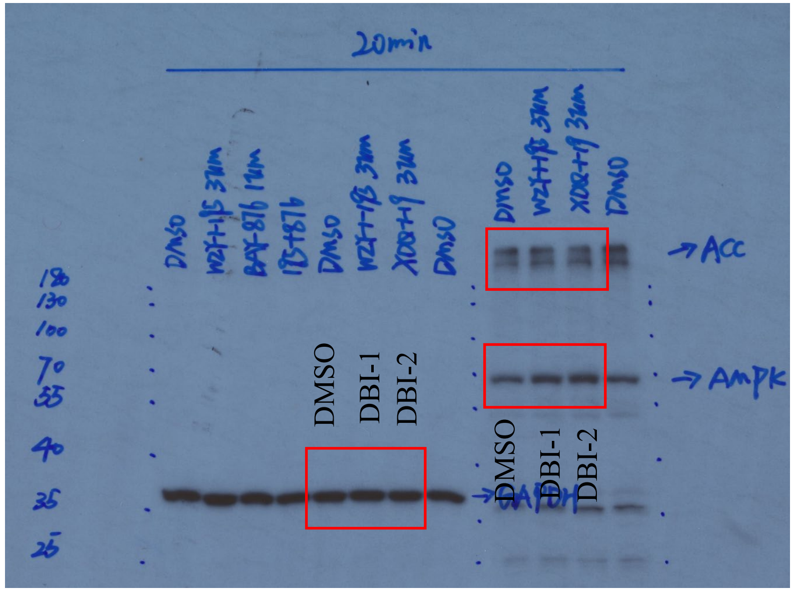

p-P70S6K
70,85 kDa

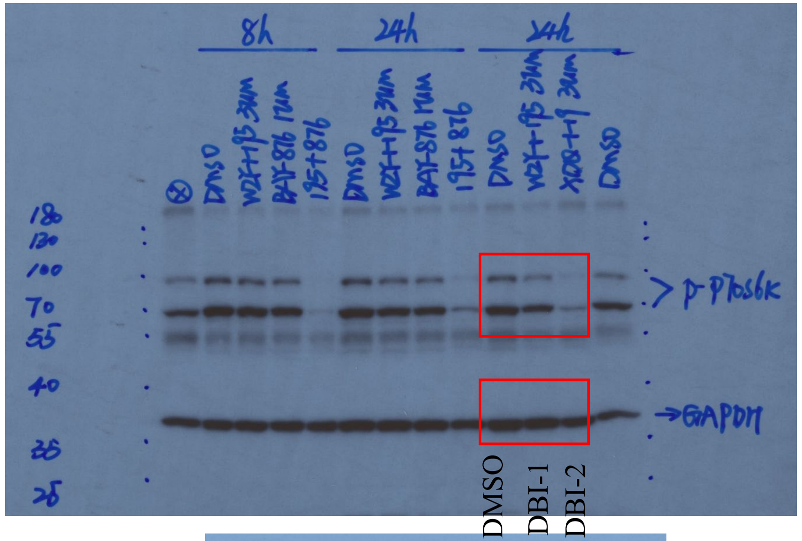

P70S6K
70,85 kDa

GAPDH
37 kDa

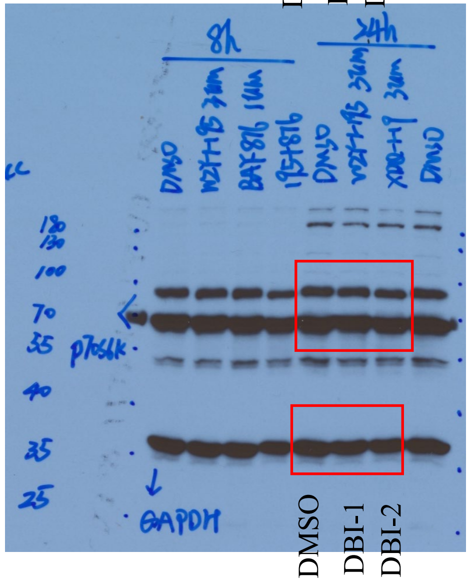

GAPDH
37 kDa

p-S6 32 kDa

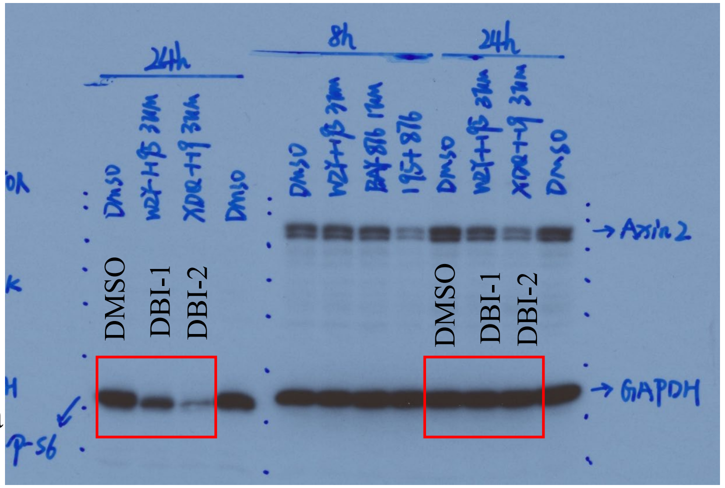

S6 32 kDa

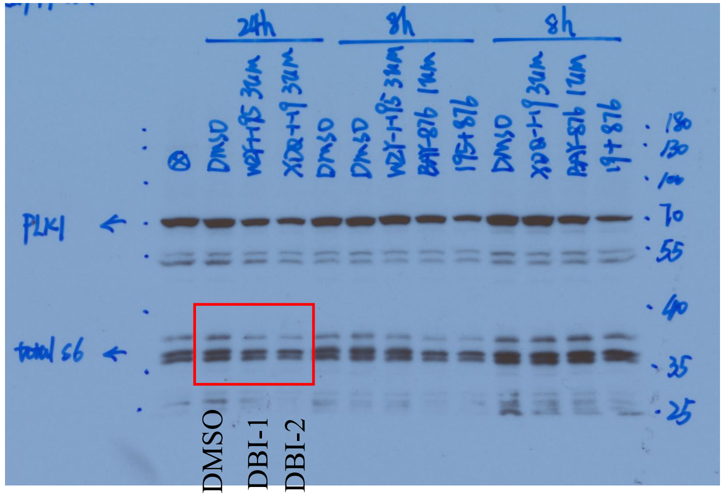

Axin2
95,98 kDa

C-Myc
57-65 kDa

GAPDH
37 kDa

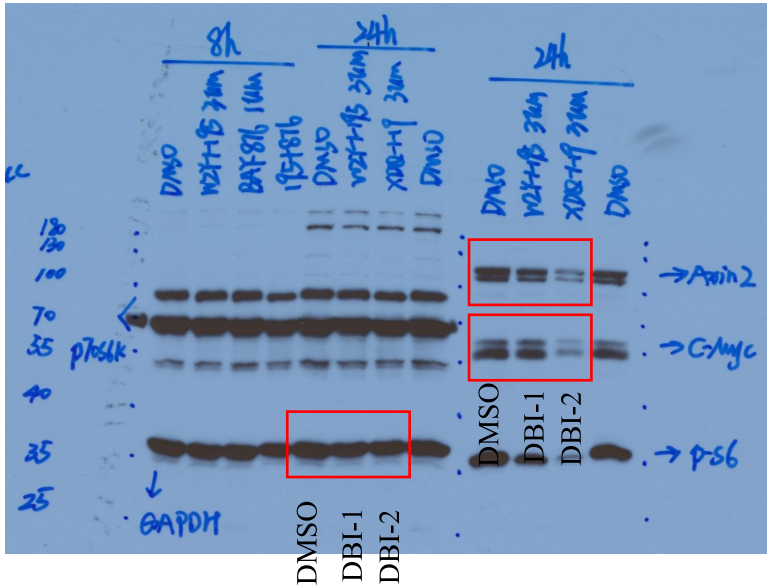

Figure 2.
(LS174T cell)

p-ACC 280 kDa

p-AMPK 62 kDa

GAPDH 37 kDa

ACC 280 kDa

AMPK 62 kDa

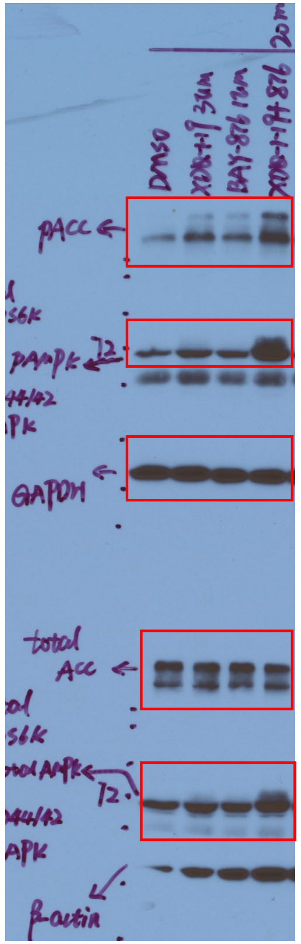

DMSO
DBI-2
BAY-876
DBI-2+BAY-876

p-P70S6K 70,85 kDa

GAPDH 37 kDa

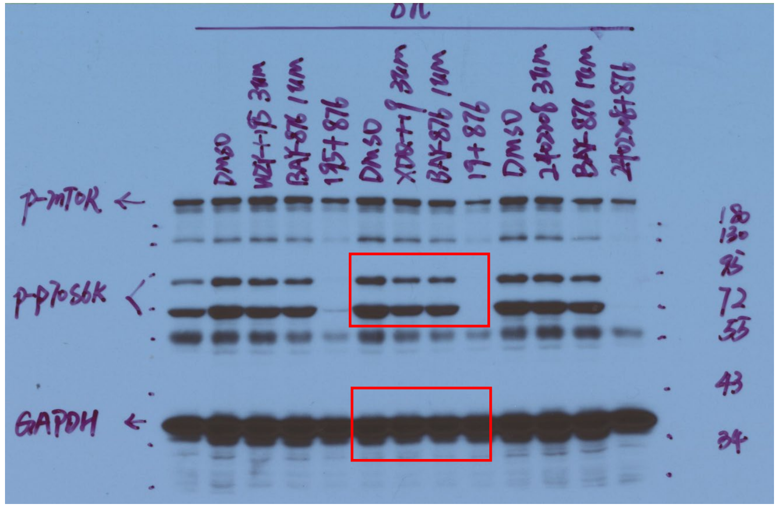

DMSO
DBI-2
BAY-876
DBI-2+BAY-876

P70S6K 70,85 kDa

GAPDH 37 kDa

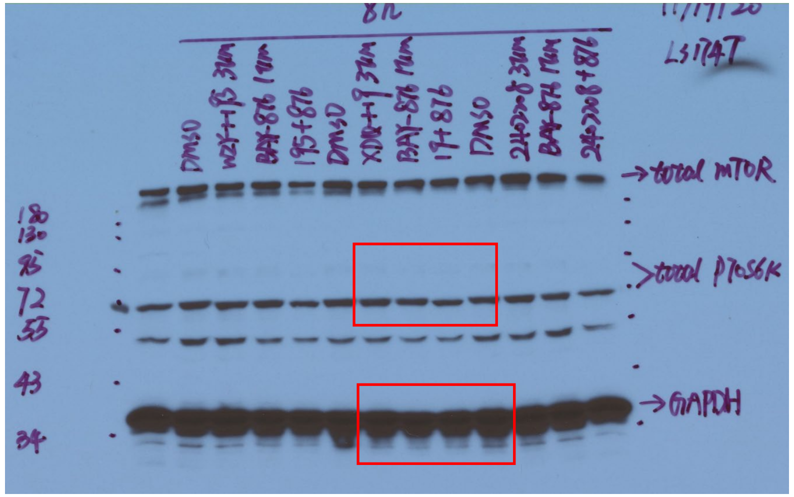

DMSO
DBI-2
BAY-876
DBI-2+BAY-876

p-S6
32 kDa

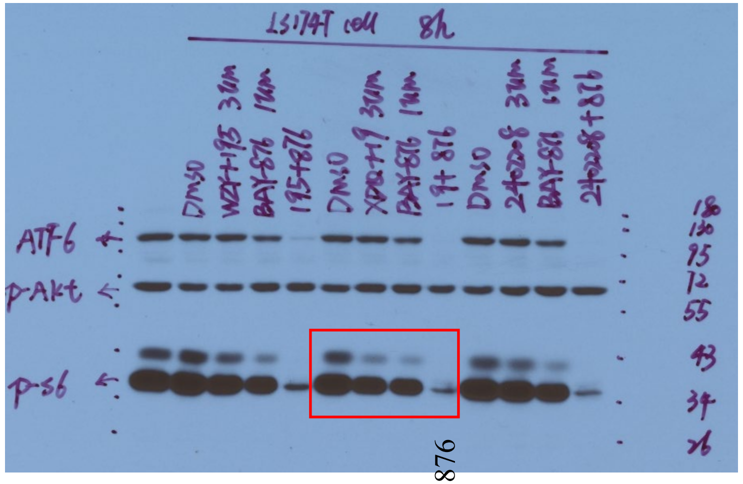

DMSO
DBI-2
BAY-876
DBI-2+BAY-876

Axin2
95,98 kDa

C-Myc
57-65 kDa

GAPDH
37 kDa

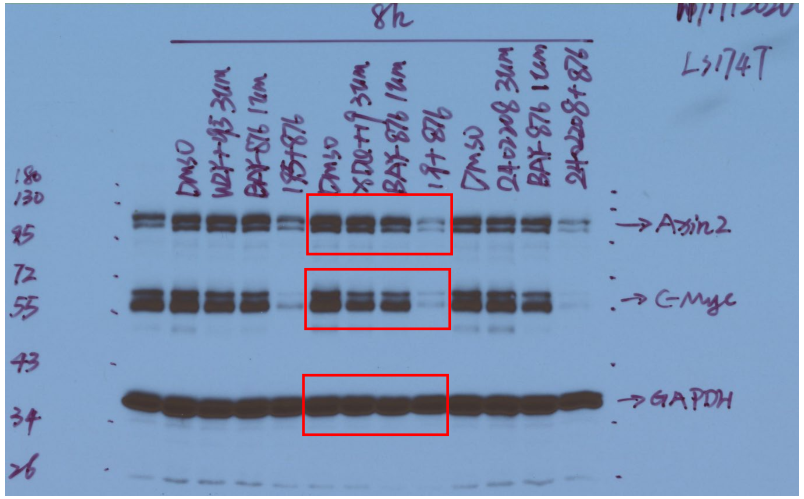

DMSO
DBI-2
BAY-876
DBI-2+BAY-876

GAPDH
37 kDa

S6
32 kDa

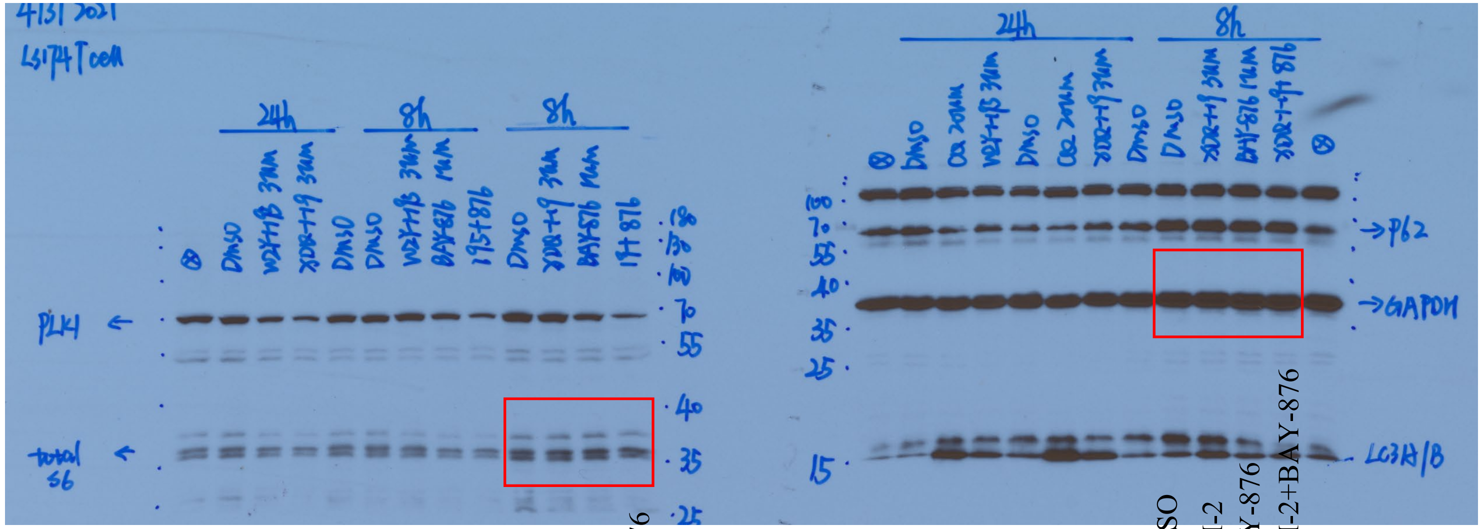

DMSO
DBI-2
BAY-876
DBI-2+BAY-876

DMSO
DBI-2
BAY-876
DBI-2+BAY-876

Figure 2.
(HCT116 cell)

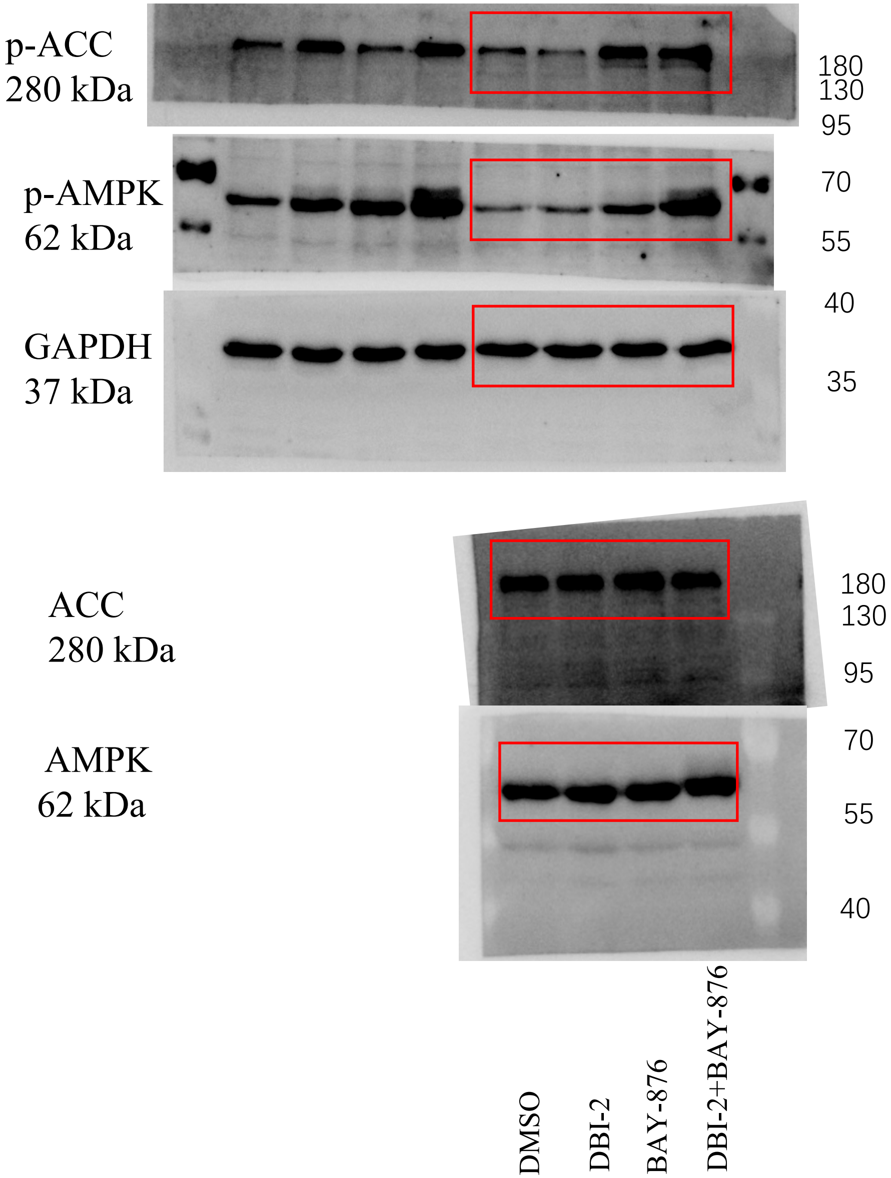

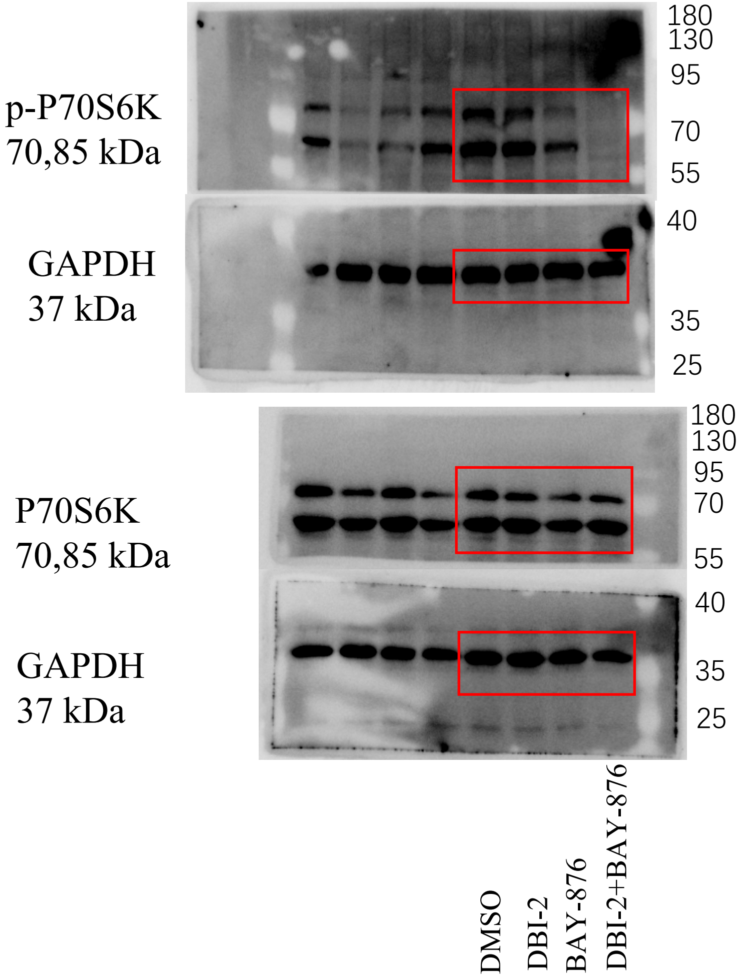

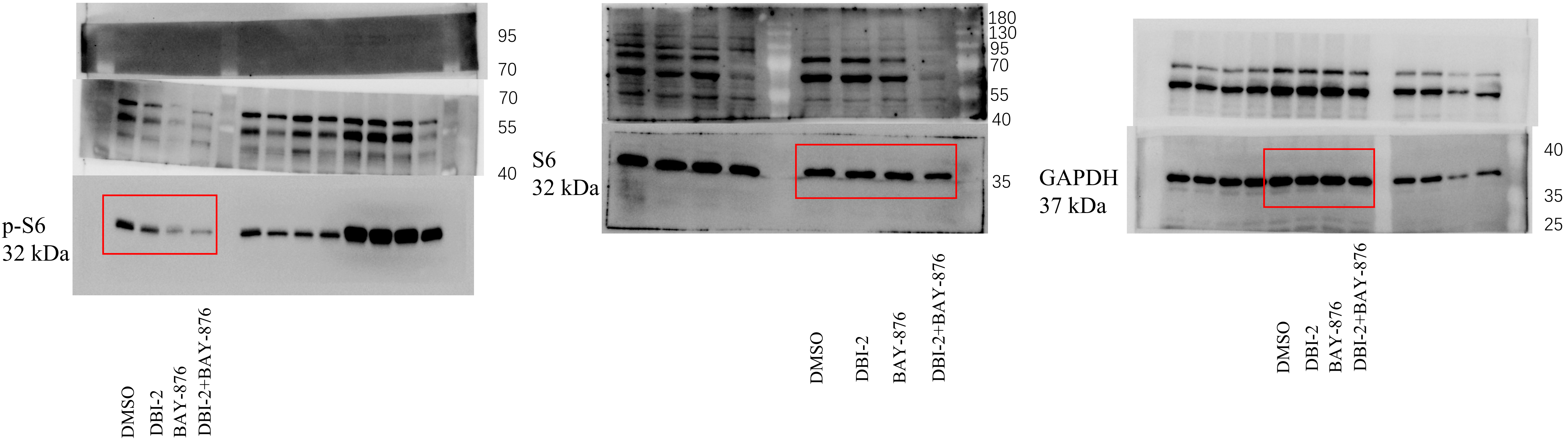
95
70
70
55
40
p-S6
32 kDa
DMSO
DBI-2
BAY-876
DBI-2+BAY-876
180
130
95
70
55
40
S6
32 kDa
35
DMSO
DBI-2
BAY-876
DBI-2+BAY-876
GAPDH
37 kDa
40
35
25
DMSO
DBI-2
BAY-876
DBI-2+BAY-876

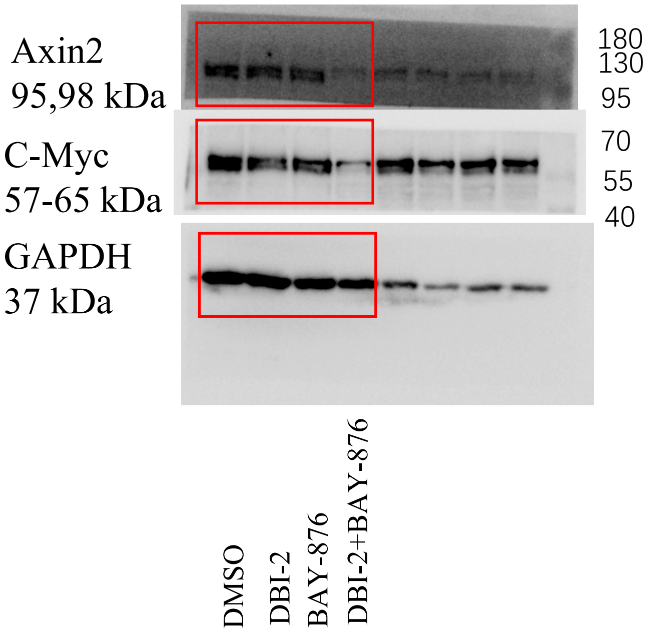
Axin2
95,98 kDa
C-Myc
57-65 kDa
GAPDH
37 kDa
180
130
95
70
55
40
DMSO
DBI-2
BAY-876
DBI-2+BAY-876
