# Supplementary material for: Inhibition of Carbohydrate Metabolism Potentiated by the Therapeutic Effects of Oxidative Phosphorylation Inhibitors in Colon Cancer Cells
Source: Cancers (Basel). 2024 Apr 2;16(7):1399. doi: 10.3390/cancers16071399 (PMC11010912; doi:10.3390/cancers16071399)
Supplement: Supplementary file 1 [file cancers-16-01399-s001.zip › cancers-2876730-supplementary.pdf]

Supplementary file

Inhibition of Carbohydrate Metabolism Potentiated by the Therapeutic Effects of Oxidative Phosphorylation Inhibitors in Colon Cancer Cells

Lichao Guo [1,2,3,†], Baochen Zhang [1,2,3,†], Wen Zhang [2,3], Yanqi Xie [2,3], Xi Chen [1,2,3], Xueke Sun [1], David S. Watt [2,3], Chunming Liu [2,3], H. Peter Spielmann [2,3] and Xifu Liu [1,*]

[1] Ministry of Education Key Laboratory of Molecular and Cellular Biology, Hebei Key Laboratory and Center for Drug Innovation and Discovery, College of Life Sciences, Hebei Normal University, Shijiazhuang 050024, China

[2] Department of Molecular and Cellular Biochemistry, College of Medicine, University of Kentucky, Lexington, KY 40536, USA

[3] Lucille Parker Markey Cancer Center, University of Kentucky, Lexington, KY 40536, USA

* Correspondence: xfliu@hebtu.edu.cn

† These authors contributed equally to this work.

This file includes:

1. **Supplementary File S1: Chemical synthesis and characterization.**
2. **Supplementary Figure S1: DBI-2 and BAY-876 synergically inhibited CRC cells in vitro.**

1. Supplementary File S1: Chemical synthesis and characterization.

General Methods. Chemicals and solvents were obtained from Sigma Aldrich (St. Louis, MO, USA) with the exception of resorcinol and 4-bromophenylacetic acid (Acros Organic, Carlsbad, CA, USA), 1,4-dibromobutane (Oakwood Chemical, Estill, SC, USA), 1,8-sibromooctane (Rhawn, Shanghai, China), sodium iodide (Fisher Scientific, Waltham, MA, USA), Piperazine, 1-(2-hydroxyethyl)piperazine (J&K Scientific, Shijiazhuang, Hebei, China, Nuclear magnetic resonance spectra were determined in $CDCl_3$, DMSO-d_6 using Bruker instruments (^1H, 600; ^{13}C, 151Mz; Bruker, Inc., Billerica, MA, USA). High resolution electrospray ionization (ESI) mass spectra were recorded on a LTQ-Orbitrap Velos mass spectrometer (Thermo Fisher Scientific, Waltham, MA, USA). The FT resolution was set at 100,000 (at 400 m/z). Samples were introduced through direct infusion using a syringe pump with a flow rate of 5 μL/min.

General Procedure A

To a mixture of resorcinol (15 mmol, 1 equiv.) and the corresponding phenylacetic acid (16.5 mmol, 1.1 eq) was added dropwise and slowly boron tribromide etherate (120 mmol, 8 eq). The mixture was heated at 85°C for 4 h under a nitrogen atmosphere. The solution was cooled to 0°C, and DMF (15 mL) was added dropwise. In a separate flask, phosphorous pentachloride (22.5 mmol, 1.5 eq) was added slowly to DMF (20 mL) at 0°C. The second mixture was warmed to 25°C and stirred for 30 min and was subsequently heated for 30 min at 55°C. The second mixture was cooled to 25°C and was added slowly to the first mixture, and the combined mixtures were stirred for 3 h at 25°C. The combined mixtures were subsequently poured into boiling 0.1M hydrochloric acid solution (120 mL). This final mixture was cooled, the precipitate was collected by filtration and washed extensively with water. The crude product was dried under reduced pressure for 12 h and purified by silica gel chromatography using 1:6 ethyl acetate: hexanes-1:6 to secure) to produce either compound **3** or **4** in Fig, 1B.

General Procedure B

To the isoflavonoid (either **3** or **4** in Fig. 1B) was added in succession DMF (40 mL), a suitable dibromoalkane (1.88 mmol, 5 eq), and potassium carbonate (18.8 mmol, 2.5 eq). The mixture was heated at 80°C for 3 h under a nitrogen atmosphere, cooled to 25°C and poured into water (100 mL) to form solid precipitate. The precipitate was collected by filtration and washed with water. The crude product was dried under

reduced pressure for 12 h and purified by silica gel chromatography using 1:2 ethyl acetate: hexanes to secure either compound **5** or **6** in Fig. 1B.

General Procedure C

To the isoflavonoid (either **5** or **6** in Fig. 1B) was added sodium iodide (2.4 mmol, 1.2 eq), DMF (18 mL), a suitable amine (2.4 mmol, 1.2 eq) for coupling to the isoflavonoid and DIPEA (7 mmol, 3.5 eq). The mixture was heated at 60°C for 3 h under a nitrogen atmosphere, quenched with water (50 mL), and extracted three times with ethyl acetate (3 x 50 mL). The organic layer was collected and dried over anhydrous magnesium sulfate, filtered, and concentrated on a rotary evaporator. The crude product was purified by silica gel chromatography using 1:10:100 ammonium hydroxide solution: methanol: dichloromethane to secure DBI-1 and DBI-2.

Compound characterization

3-(4-Bromophenyl)-7-hydroxy-4H-chromen-4-one (3). Following general procedure A, **3** was obtained from **1** in 39% yield. ^{1}H NMR (600 MHz, DMSO) δ 10.86 (s, 1H), 8.45 (s, 1H), 7.99 (d, *J* = 8.8 Hz, 1H), 7.72 – 7.60 (m, 2H), 7.59 – 7.39 (m, 2H), 6.96 (dd, *J* = 8.8, 2.2 Hz, 1H), 6.90 (d, *J* = 2.2 Hz, 1H). ^{13}C NMR (151 MHz, DMSO) δ 174.62, 163.24, 157.93, 154.57, 131.87, 131.53, 131.45, 127.80, 122.84, 121.54, 116.98, 115.86, 102.69.

3-(3,4-Dichlorophenyl)-7-hydroxy-4H-chromen-4-one (4). Following general procedure A, **4** was obtained from **2** in 24% yield. ^{1}H NMR (600 MHz, DMSO) δ 10.91 (s, 1H), 8.53 (s, 1H), 7.99 (d, *J* = 8.7 Hz, 1H), 7.89 (s, 1H), 7.70 (d, *J* = 8.3 Hz, 1H), 7.61 (d, *J* = 8.2 Hz, 1H), 6.97 (d, *J* = 8.6 Hz, 1H), 6.91 (s, 1H). ^{13}C NMR (126 MHz, cdcl$_3$) δ 181.85, 170.73, 165.28 162.63, 140.69, 138.63, 138.42, 138.20, 138.10, 136.89, 135.19, 128.96, 124.25, 123.36, 110.11.

7-(4-Bromobutoxy)-3-(4-bromophenyl)-4H-chromen-4-one (5). Following general procedure B, **5** was obtained from **3** and 1,4-dibromobutane in 44% yield. ^{1}H NMR (500 MHz, DMSO) δ 8.51 (s, 1H), 8.01 (d, *J* = 8.9 Hz, 1H), 7.62 (d, *J* = 8.4 Hz, 2H), 7.54 (d, *J* = 8.3 Hz, 2H), 7.18 (d, *J* = 1.9 Hz, 1H), 7.08 (dd, *J* = 8.8, 1.9 Hz, 1H), 4.16 (t, *J* = 6.1 Hz, 2H), 3.61 (t, *J* = 6.6 Hz, 3H), 2.04 – 1.93 (m, 2H), 1.92 – 1.83 (m, 2H).

^{13}C NMR (100 MHz, DMSO) δ 174.66 (s), 163.60, 157.91, 154.88, 131.70, 131.55, 131.43, 127.43, 123.05, 121.63, 117.94, 115.71, 101.63, 68.15, 35.22, 29.42, 27.56.

7-(2-Bromoethoxy)-3-(3,4-dichlorophenyl)-4H-chromen-4-one (6). Following general procedure B, **6** was obtained from **4** and 1,2-dibromoethane in 32% yield. ^{1}H NMR (600 MHz, DMSO) δ 8.70 (s, 1H), 8.12 (d, *J* = 8.9 Hz, 1H), 7.98 (d, *J* = 2.0 Hz, 1H), 7.78 (d, *J* = 8.4 Hz, 1H), 7.69 (dd, *J* = 8.4, 2.0 Hz, 1H), 7.33 (d, *J* = 2.4 Hz, 1H), 7.22 (dd, *J* = 8.9, 2.4 Hz, 1H), 4.58 (t, 2H), 3.94 (t, 2H). ^{13}C NMR (126 MHz, DMSO) δ 177.14, 165.62, 160.43, 158.29, 135.74, 133.92, 133.65,133.56, 133.39, 132.15, 130.23, 124.47, 120.84, 118.44, 104.66, 71.70, 34.05.

3-(4-bromophenyl)-7-(2-((2-(dimethylamino)ethyl)(methyl)amino)ethoxy)-4H-chromen-4-one (DBI-1). Following general procedure c, DBI-1 was obtained from **5** and N^{1},N^{1},N^{2}-trimethylethane-1,2-diamine in 36% yield. ^{1}H NMR (500 MHz, DMSO) δ 8.51 (s, 1H), 8.01 (d, *J* = 8.9 Hz, 1H), 7.65 – 7.59 (m, 2H), 7.57 – 7.51 (m, 2H), 7.16 (d, *J* = 2.3 Hz, 1H), 7.07 (dd, *J* = 8.9, 2.4 Hz, 1H), 4.14 (t, *J* = 6.5 Hz, 2H), 2.56 (dd, *J* = 17.5, 5.2 Hz, 4H), 2.46 (d, *J* = 7.4 Hz, 2H), 2.31 (s, 6H), 2.22 (s, 3H), 1.82 – 1.69 (m, 2H), 1.65 – 1.51 (m, 2H). ^{13}C NMR (126 MHz, DMSO) δ 177.27, 166.34, 160.56, 157.51, 134.33, 134.18, 134.06, 130.06, 125.67, 124.26, 120.52, 118.34, 104.22, 71.50, 59.75, 58.57, 56.74, 29.24, 25.74.

3-(3,4-Dichlorophenyl)-7-(2-(4-(2-hydroxyethyl)piperazin-1-yl)ethoxy)-4H-chromen-4-one (DBI-2). Following general procedure C, DBI-2 was obtained from **6** and 2-(piperazin-1-yl)ethan-1-ol in 20% yield. ^{1}H NMR (600 MHz, $CDCl_3$) δ 8.22 (d, J = 8.9 Hz, 1H), 7.97 (s, 1H), 7.59 (d, J = 8.3 Hz, 2H), 7.48 (d, J = 8.3 Hz, 2H), 7.02 (dd, J = 8.9, 1.9 Hz, 1H), 6.87 (d, J = 1.7 Hz, 1H), 4.08 (t, J = 6.4 Hz, 2H), 3.63 (t, J = 5.2 Hz, 2H), 2.92 – 2.41 (m, 10H), 2.36 (t, 2H), 1.93 – 1.77 (m, 2H), 1.56 – 1.46 (m, 4H), 1.45 – 1.31 (m, 6H). ^{13}C NMR (151 MHz, $CDCl_3$) δ 175.05, 163.42, 157.88, 152.82, 132.60, 132.34, 131.95, 130.70, 130.41, 128.25, 127.84, 123.33, 118.30, 115.27, 100.93, 66.77, 59.14, 57.71, 56.84, 53.78, 52.75 (s).

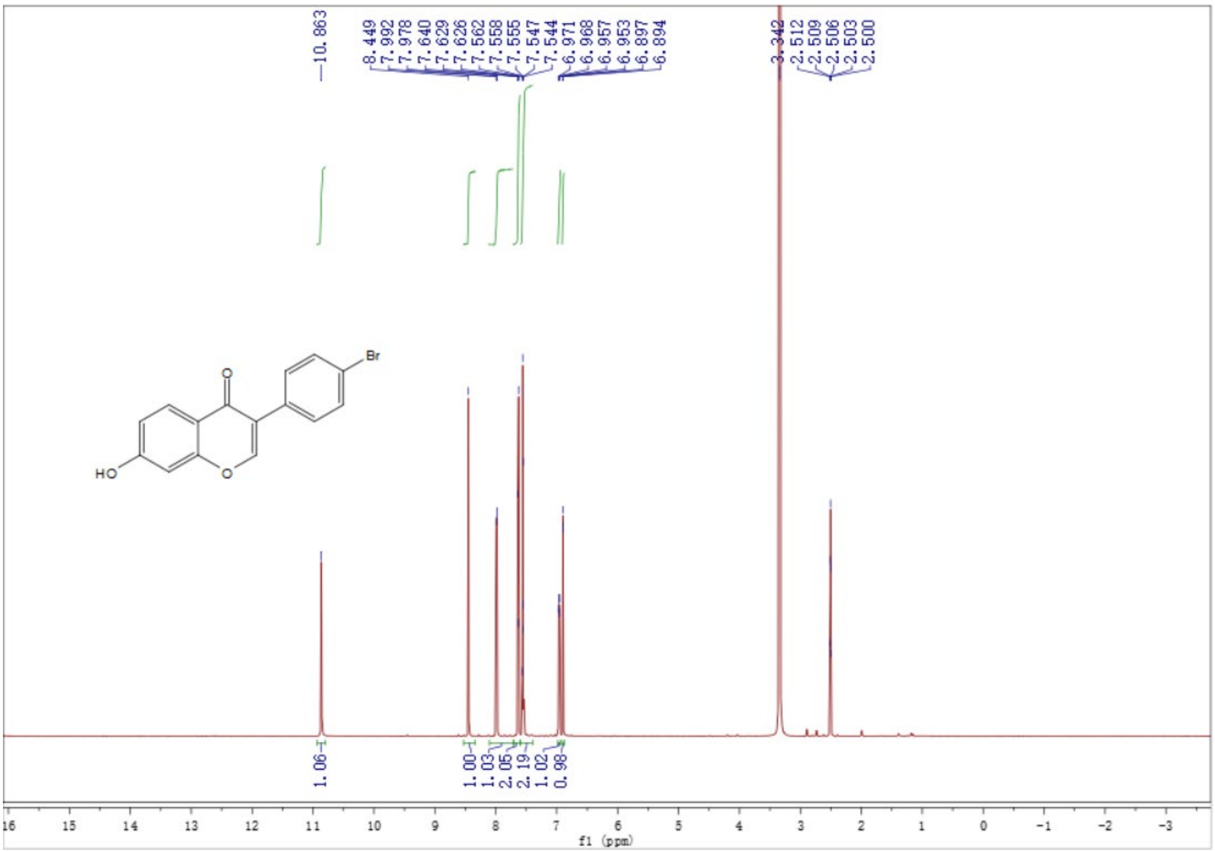

3 NMR ^{1}H

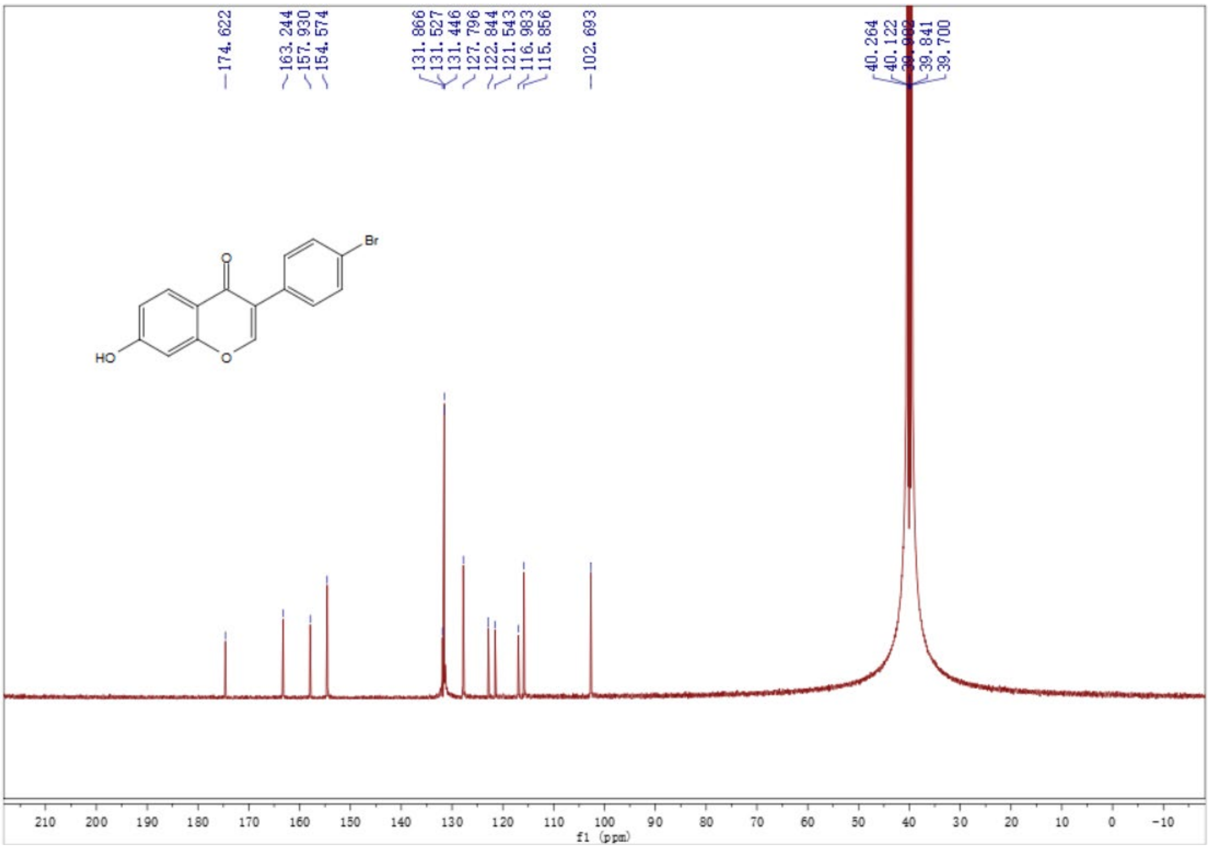

3 NMR ^{13}C

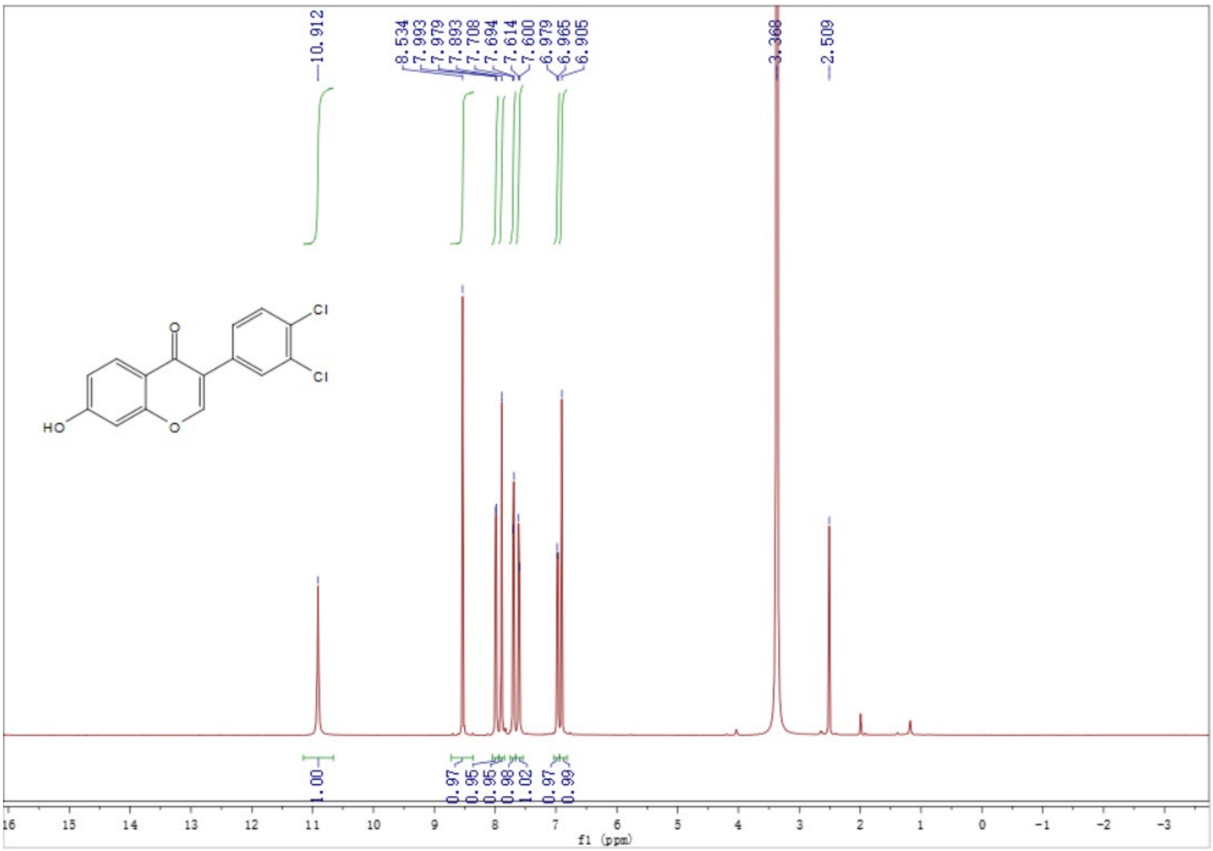

4 NMR ^{1}H

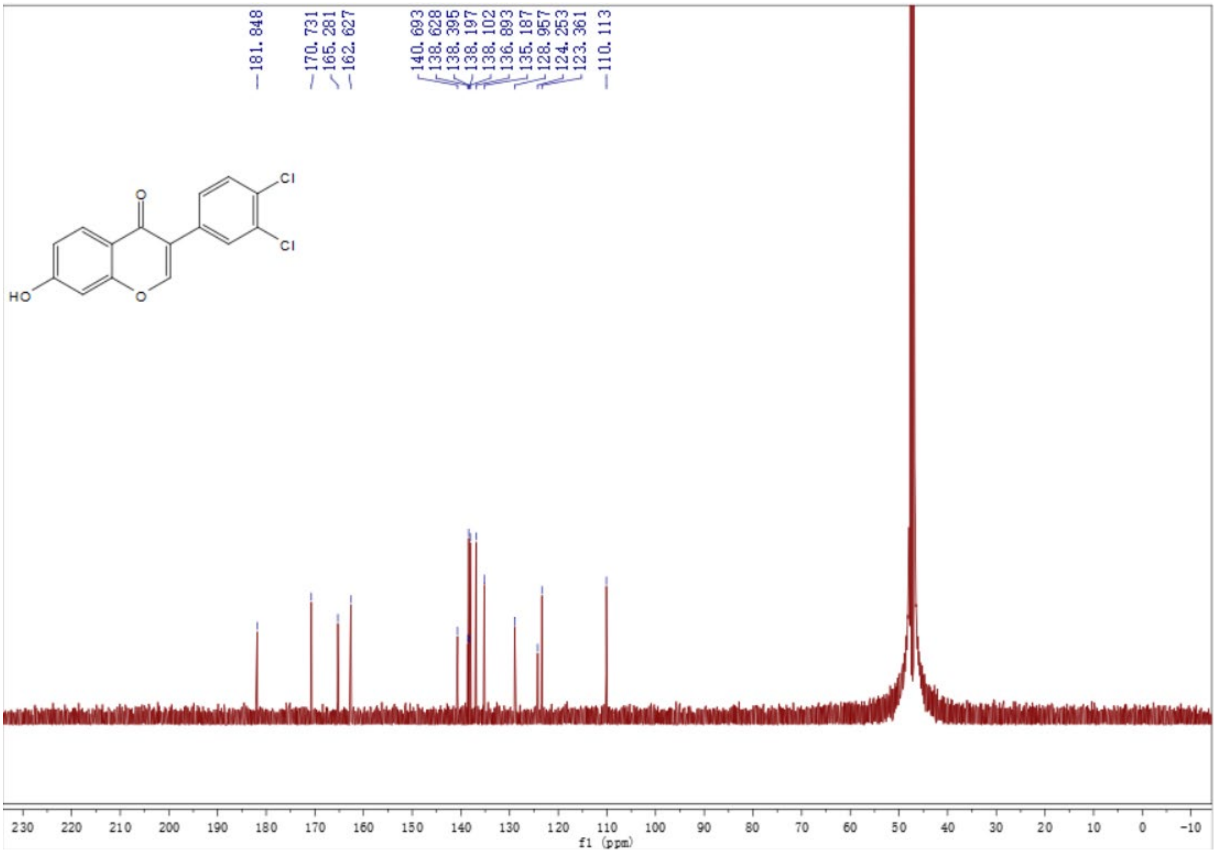

4 NMR ^{13}C

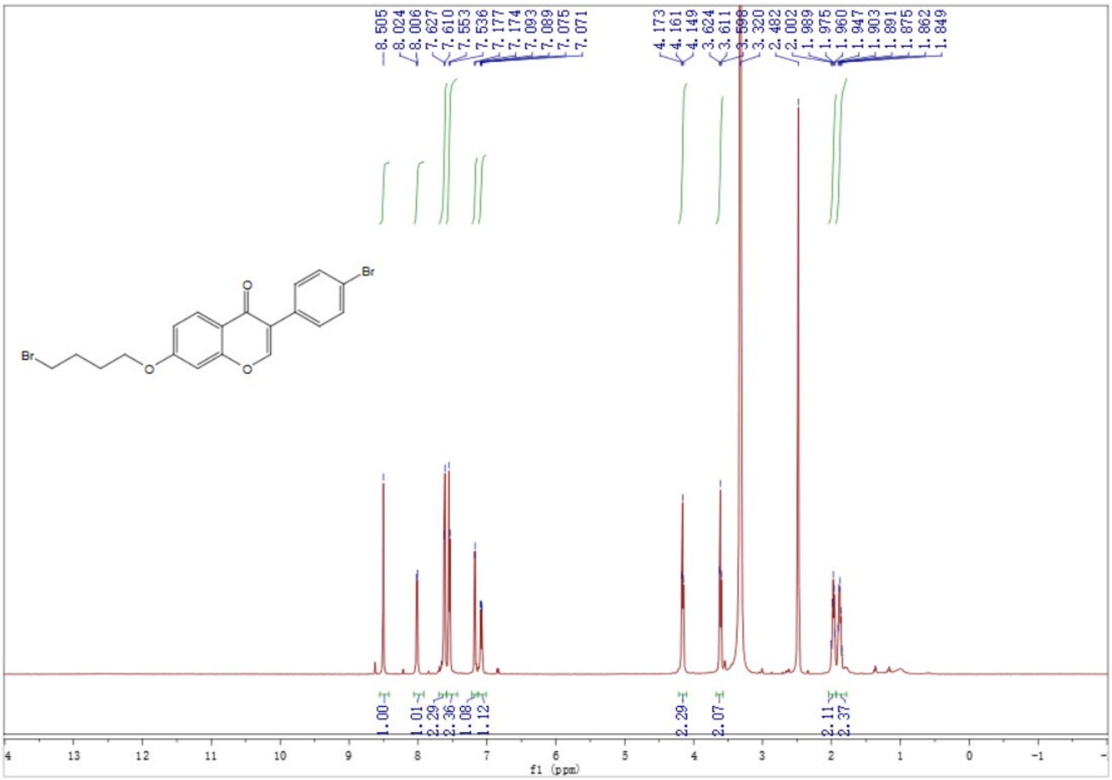

5 NMR ^{1}H

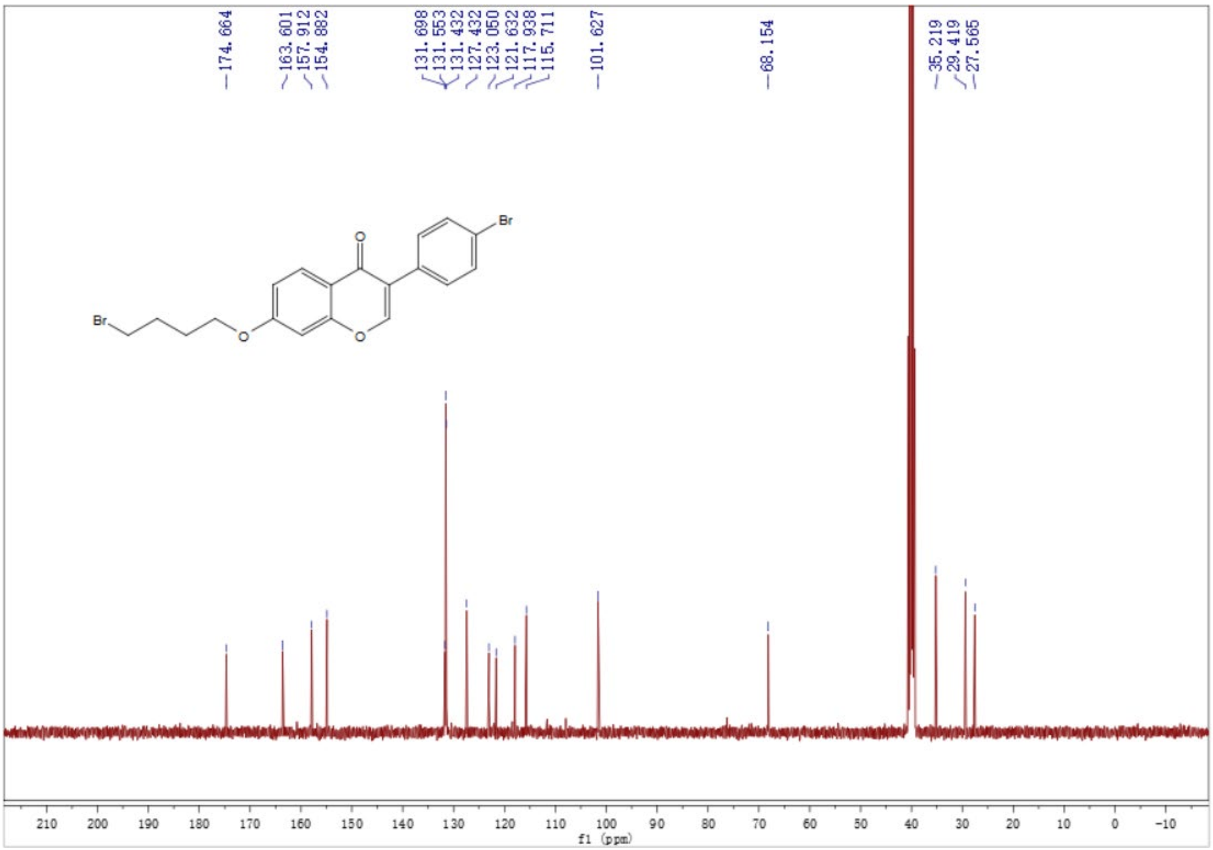

5 NMR ^{13}C

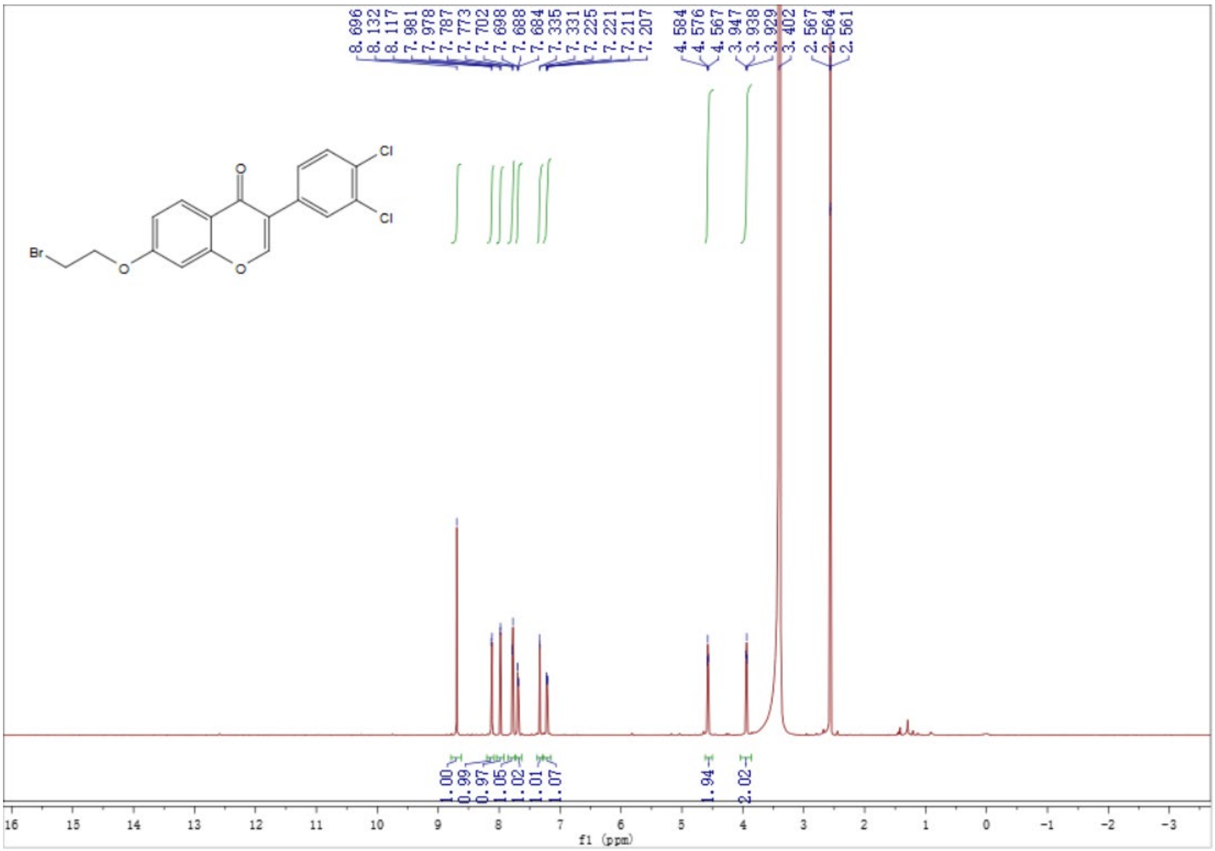

6 NMR ^{1}H

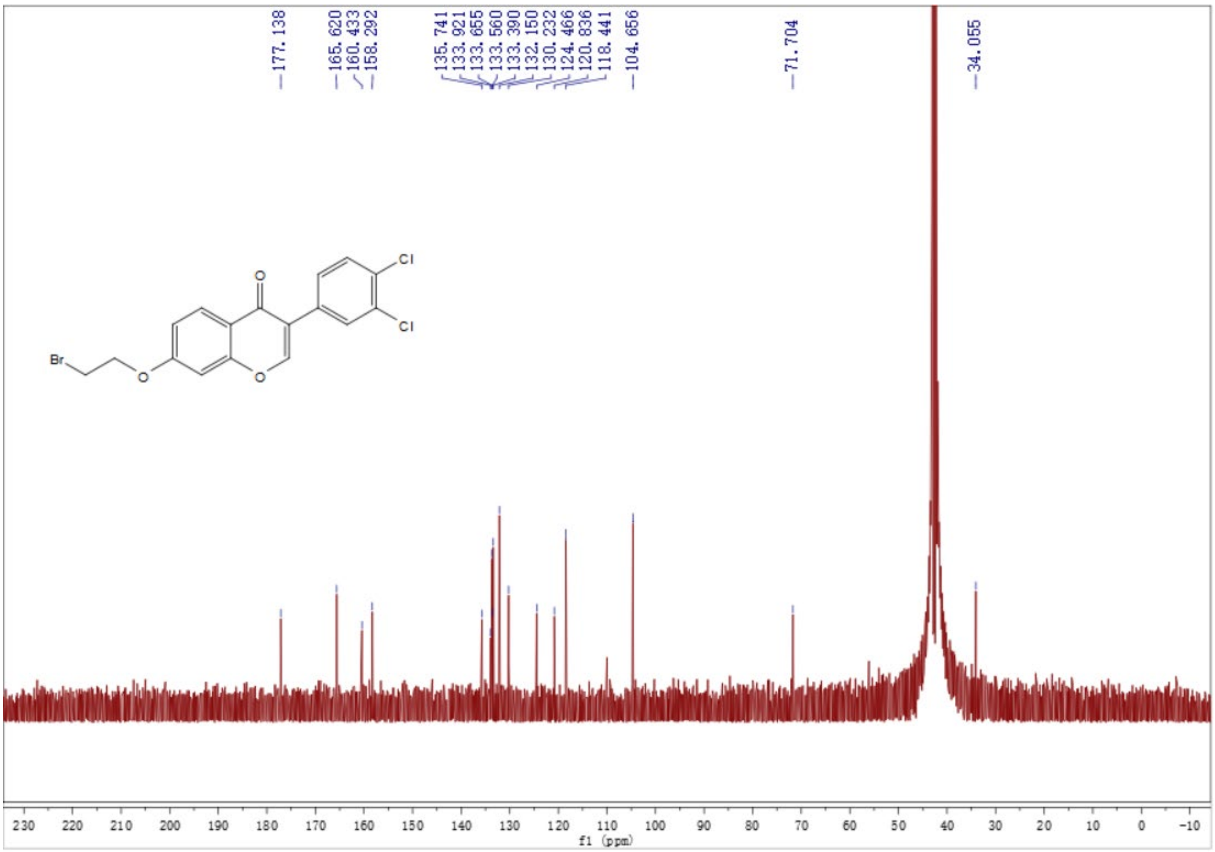

6 NMR ^{13}C

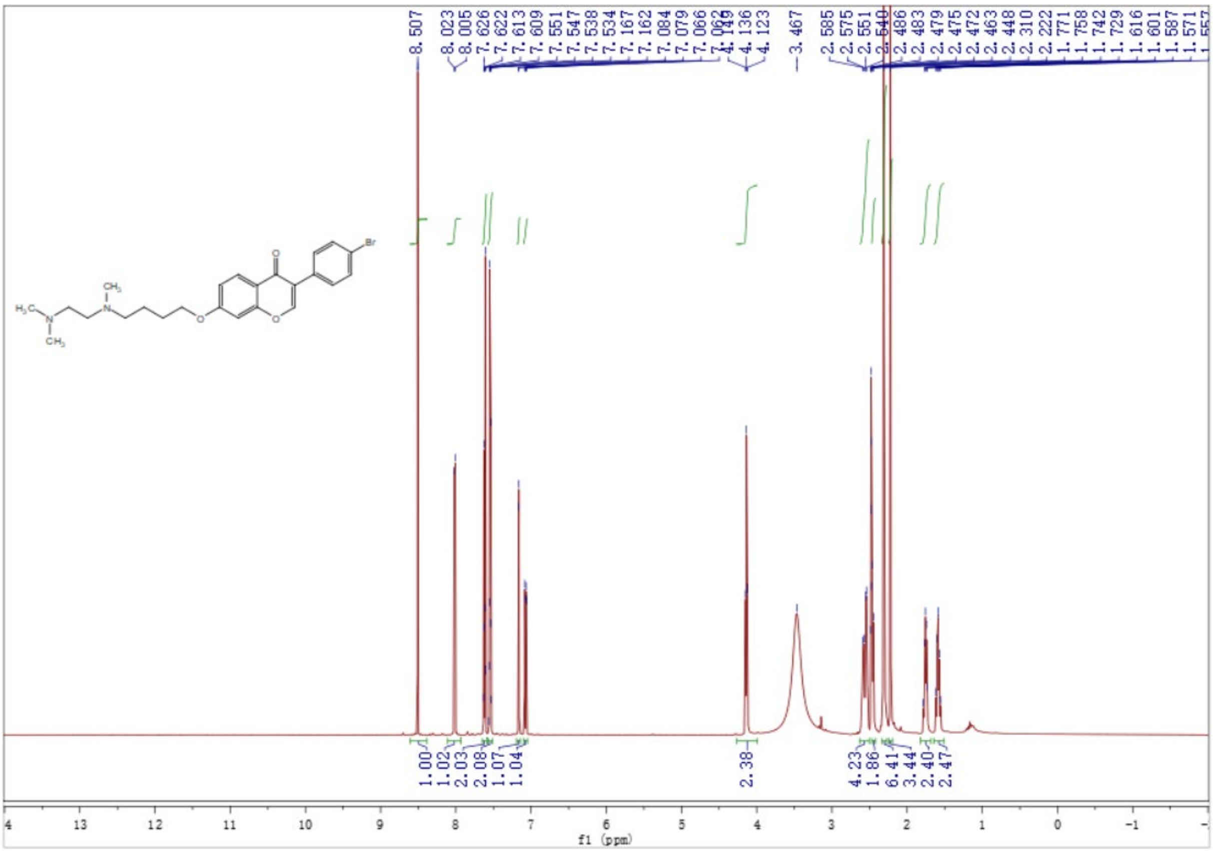

DBI-1 NMR ^{1}H

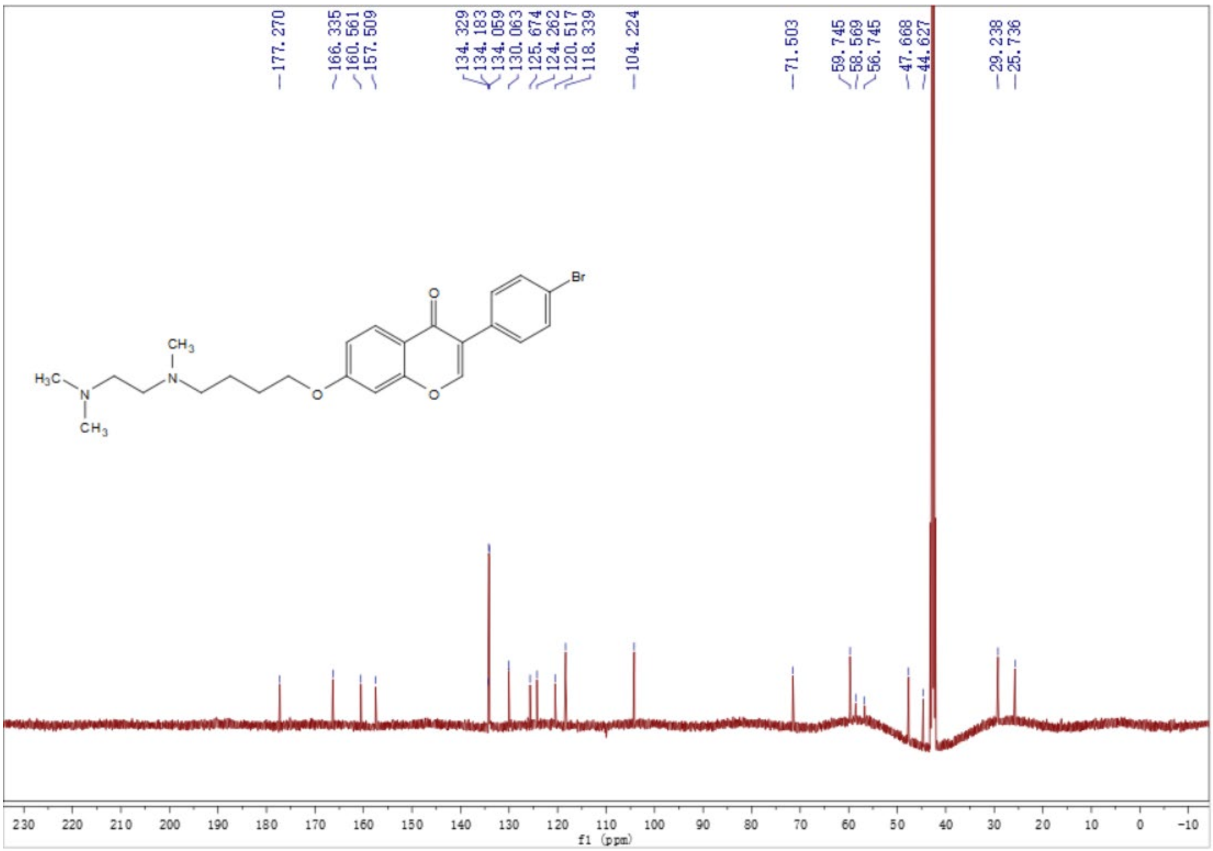

DBI-1 NMR ^{13}C

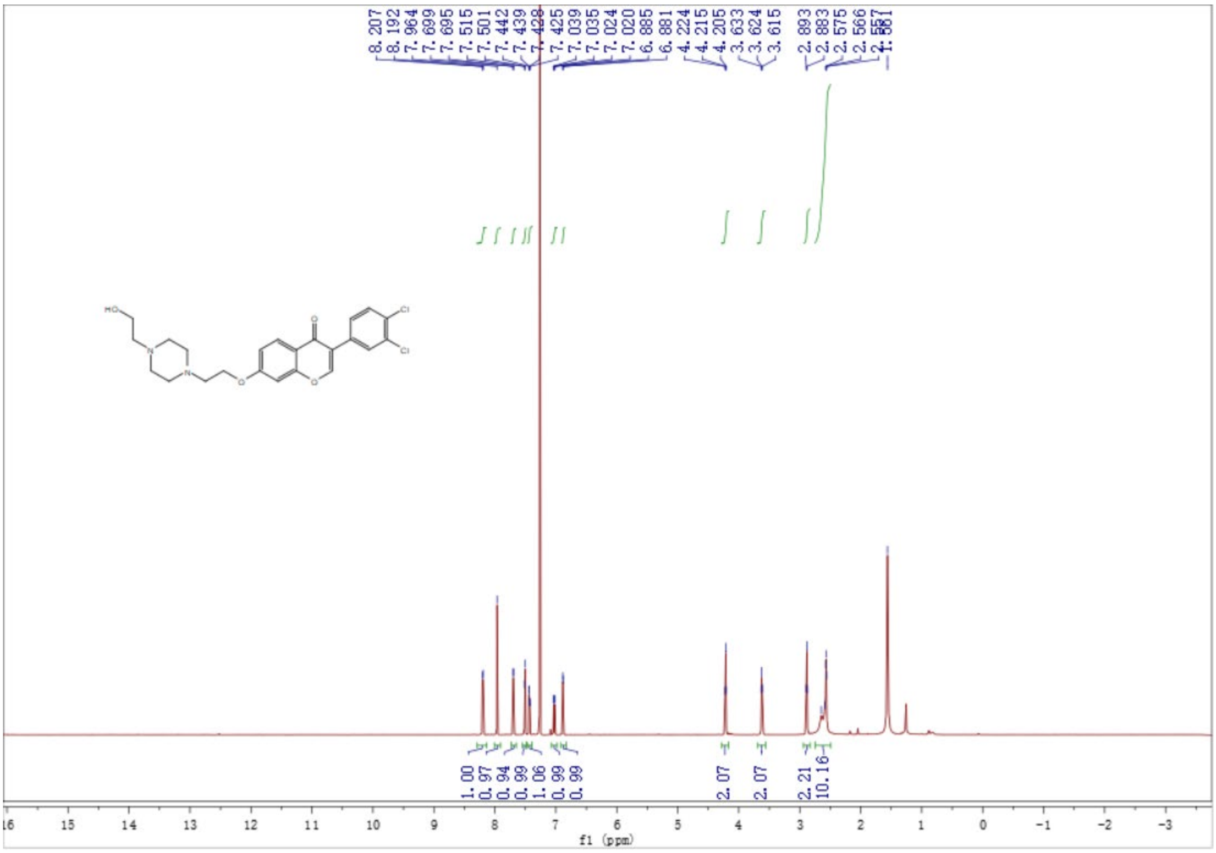

DBI-2 NMR ^{1}H

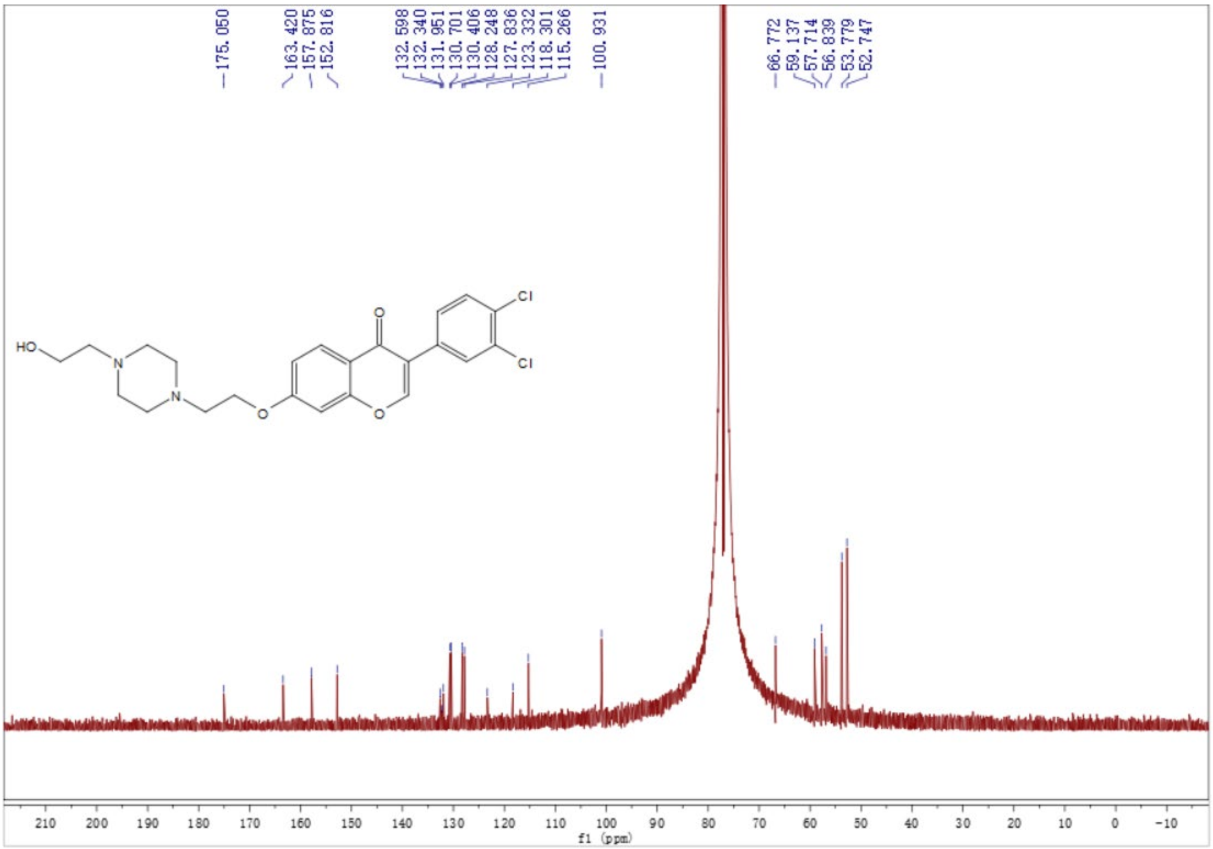

DBI-2 NMR ^{13}C

Supplementary Figure S1: DBI-2 and BAY-876 synergically inhibited CRC cells in vitro.

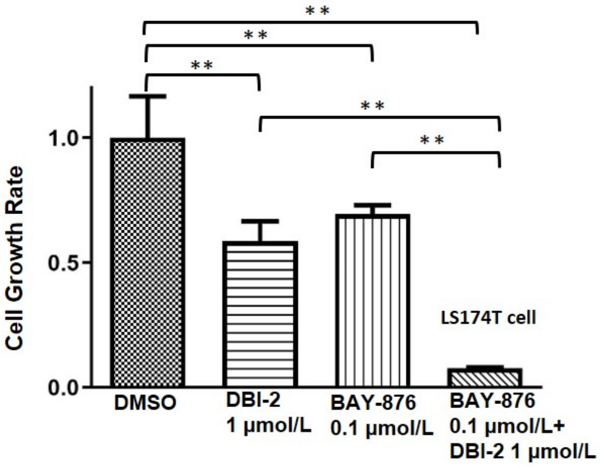

Supplementary Figure S1. DBI-2 and BAY-876 synergically inhibited CRC cells in vitro. The LS174T cells were treated with DBI-2 or BAY-876 at specified concentrations for 5 days, and the cell proliferation was determined using CCK-8. Data are presented as the means ± SEM. * $p < 0.05$, and ** $p < 0.01$, n = 3. One-way ANOVA followed by Tukey's multiple comparisons test was applied as the statistical method.
